# A Spatiotemporal Uncertainty-Aware Task Planning Framework for Cooperative Vehicle–UAV Remote Sensing Monitoring and Verification in Complex Terrain

**DOI:** 10.3390/s26175627

**Published:** 2026-09-04

**Authors:** Haoran Xu, Lei Hu, Zhiwen Lu, Xiaohui Huang, Yuewei Wang, Xiaodao Chen

**Affiliations:** School of Computer Science, China University of Geosciences, Wuhan 430078, China; xhr@cug.edu.cn (H.X.); 1202521681@cug.edu.cn (L.H.); 1202421641@cug.edu.cn (Z.L.); xhhuang@cug.edu.cn (X.H.); yuewei.w@cug.edu.cn (Y.W.)

**Keywords:** unmanned aerial vehicles (UAVs), remote sensing, natural resource survey and monitoring, task planning, spatiotemporal uncertainty

## Abstract

Unmanned aerial vehicles (UAVs) have been increasingly used as flexible sensing platforms for remote sensing applications due to their rapid deployment and efficient data acquisition capabilities. Cooperative vehicle–UAV systems have shown great potential for large-scale remote sensing monitoring and field verification. However, existing task planning methods often overlook the characteristics of remote sensing verification missions, including fragmented target parcels and spatiotemporal uncertainties caused by complex terrain, which limits scheduling efficiency and robustness. To address these challenges, this paper proposes a spatiotemporal uncertainty-aware task planning framework for vehicle–UAV cooperative remote sensing verification. The framework integrates UAV capability-constrained task region generation, terrain-driven spatial uncertainty risk classification, a dual-channel genetic algorithm (DC-GA), and an uncertainty-aware two-stage scheduling framework (UATSF). Experiments in two real-world study areas validate the effectiveness of the proposed framework. The region-merging strategy reduces total travel distance and travel time while improving UAV utilization, and DC-GA consistently reduces the system makespan across different vehicle configurations. Moreover, the two-stage strategy, which combines deterministic optimization with Monte Carlo robustness assessment, reduces planned completion time by 6.80–11.09% compared with worst-case scheduling while achieving 86.20–98.40% reliability under the modeled uncertainty and assumed simulation settings. The results demonstrate that the proposed framework improves task planning efficiency and robustness for vehicle–UAV cooperative operations in complex terrain environments.

## 1. Introduction

**Figure 1:**
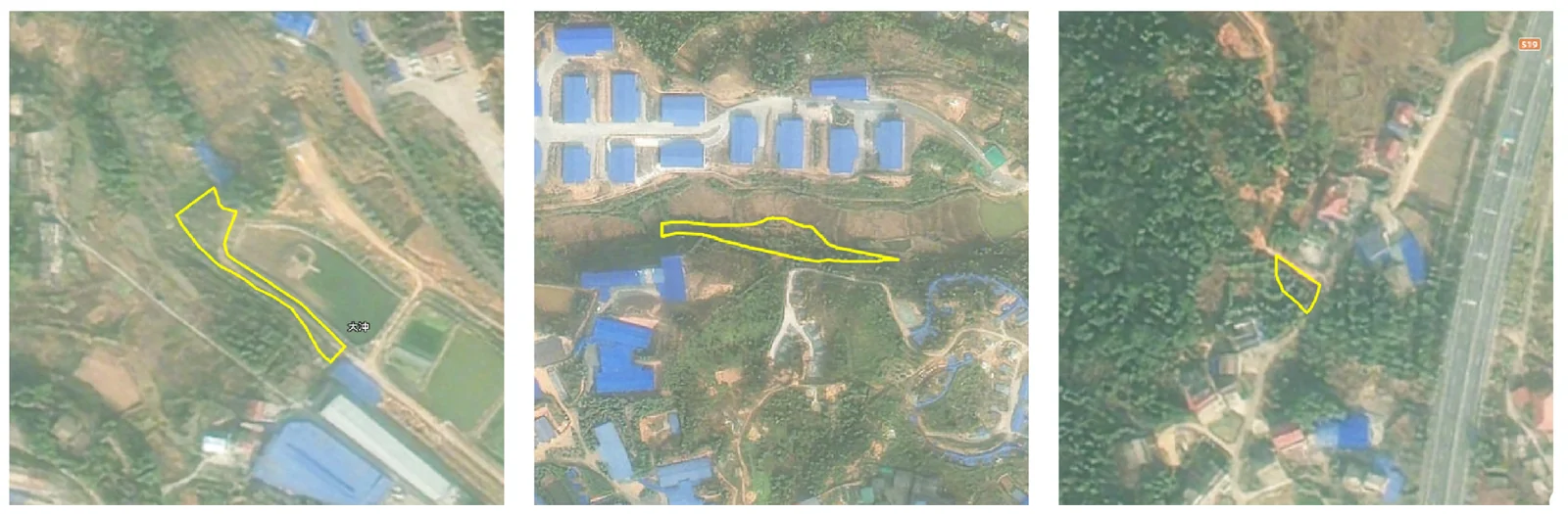
Examples of parcels requiring field verification.

Natural resource survey and monitoring have become essential technical support for cultivated land protection [1], land-use change monitoring [2], ecological environment supervision [3], and territorial spatial governance [4]. With the continuous improvement of high-resolution satellite remote sensing, large-scale image interpretation can rapidly identify areas with potential changes [5]. However, remote sensing imagery alone cannot fully replace field verification. To ensure the reliability of interpretation results, a large number of candidate parcels require on-site inspection, as illustrated in Figure 1. Field investigation is conducted by collecting on-site images, videos, and positioning information to verify the authenticity of the interpreted changes. Therefore, improving the efficiency of large-scale and multi-target remote sensing verification has become an important research topic in intelligent natural resource surveying.

**Figure 2:**
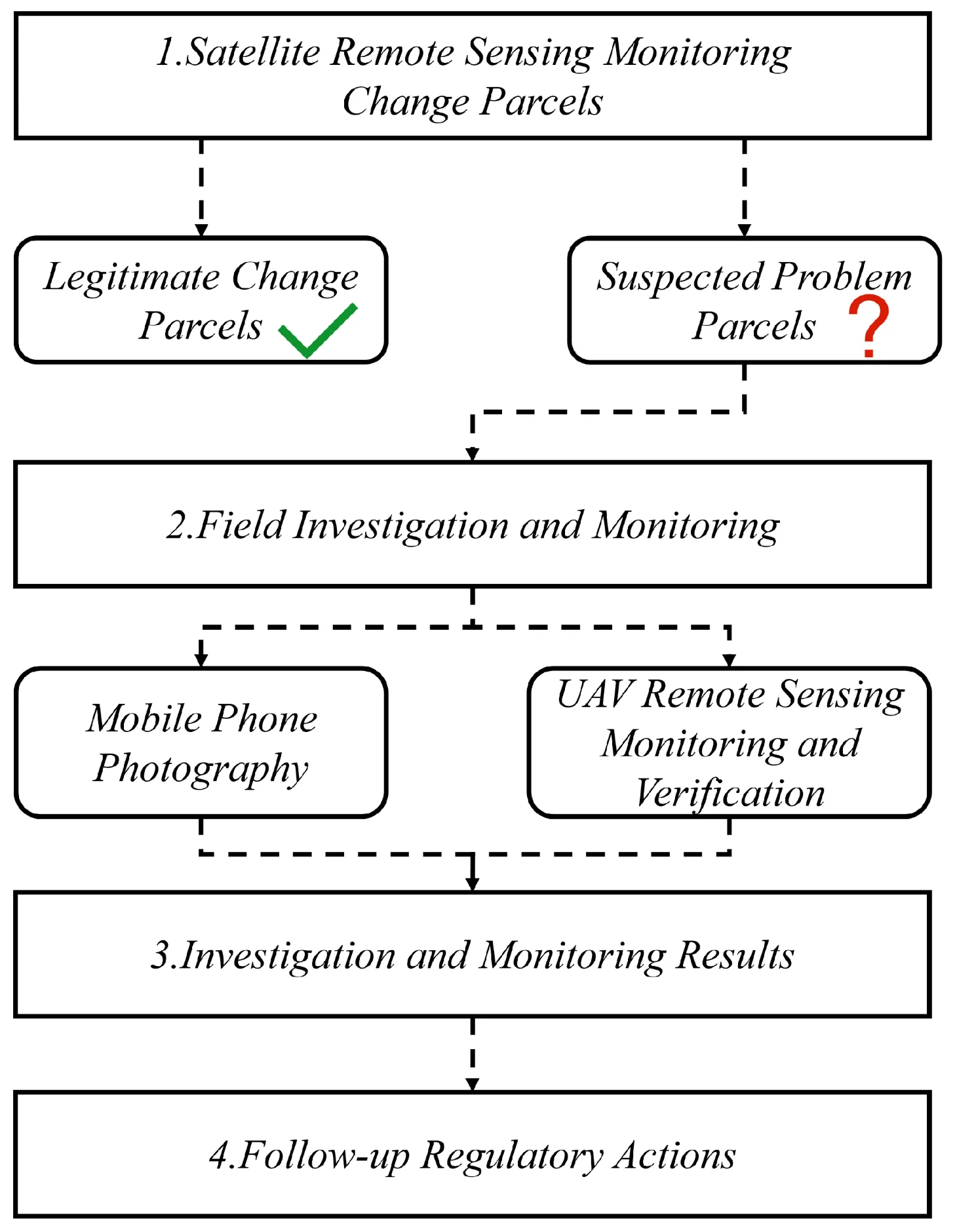
The overall workflow of natural resource monitoring and verification.

Figure 2 presents the overall workflow of natural resource monitoring and verification. Change parcels are first identified through satellite remote sensing monitoring. After legitimacy screening, suspected problem parcels are generated and forwarded to the field verification stage. According to site conditions, field verification is carried out using either mobile phone photography or UAV-based remote sensing monitoring to acquire field evidence and produce investigation and monitoring results. The resulting information is then used to support natural resource issue identification and subsequent regulatory actions.

**Figure 3:**
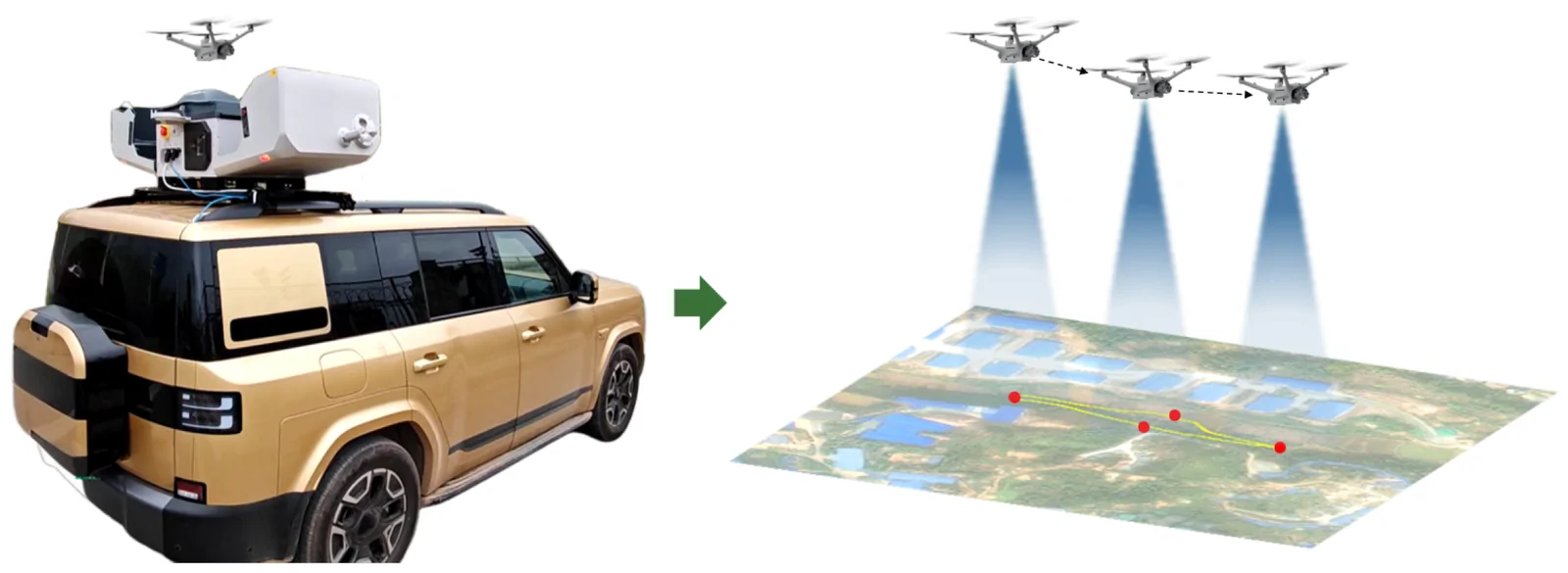
Vehicle–UAV collaborative operation system.

UAVs have become an important sensing tool for field verification in natural resource surveys due to their high mobility, flexible deployment, and high spatial resolution [6,7,8,9]. However, their operational range is constrained by limited flight endurance and communication distance [10,11]. As a result, a single UAV cannot continuously accomplish monitoring tasks over large-scale areas. To address this limitation, we previously proposed a vehicle–UAV collaborative operation system for natural resource surveys [12], as illustrated in Figure 3. In this system, a ground vehicle serves as a mobile support platform that provides UAV transportation, takeoff and landing, battery charging, and communication services, while the UAV is responsible for remote sensing data acquisition over target areas. This system overcomes the operational constraints imposed by fixed takeoff and landing sites. It also substantially improves continuous monitoring capability in complex environments, providing an efficient mobile sensing solution for large-scale natural resource surveys.

The vehicle–UAV collaborative system merely provides the operational platform for task execution, while its overall operational efficiency still depends heavily on an effective task planning strategy. Our previous work focused on the design and validation of an integrated vehicle–UAV platform. In terms of task planning, the system mainly generated a simple bow-shaped coverage path for each individual parcel, together with a nearby vehicle navigation point. However, it did not address higher-level planning requirements for large-scale verification missions, including organizing dispersed parcels under UAV capability constraints, coordinating multiple vehicle–UAV pairs, and accounting for uncertainties in vehicle travel and UAV operation times. Therefore, a more comprehensive task planning method is needed to improve both operational efficiency and scheduling robustness in large-scale vehicle–UAV remote sensing verification.

Compared with conventional vehicle routing problems, natural resource remote sensing verification tasks exhibit much more complex operational characteristics. On the one hand, the large number of geographically dispersed target parcels must first be partitioned into executable task regions according to the operational capabilities of UAVs, thereby reducing the scheduling scale while ensuring task feasibility. On the other hand, multiple vehicles and multiple UAVs are required to collaboratively accomplish region access, UAV-based data acquisition, and battery charging support. As a result, task assignment, visitation sequencing, and resource coordination are tightly coupled, giving rise to a typical large-scale combinatorial optimization problem.

Furthermore, under complex terrain conditions, UAV operation times are inherently uncertain rather than deterministic [13,14]. Random factors, including wind disturbances, GPS positioning errors, and flight-control response delays, may cause the actual operation time to deviate from its offline estimate [15]. Vehicle travel time is also subject to uncertainty due to real-world factors such as traffic conditions. Consequently, scheduling based on deterministic operation times is prone to discrepancies between planned and actual execution, thereby reducing the reliability of completing verification tasks within the required time horizon. Therefore, developing a vehicle–UAV collaborative planning method that simultaneously improves operational efficiency and execution robustness under complex terrain conditions remains a critical challenge that urgently needs to be addressed.

However, existing planning methods do not provide an integrated solution for natural resource remote sensing monitoring and verification. In particular, they rarely consider irregular parcel organization, multi-vehicle and multi-UAV scheduling, and uncertainties in actual vehicle travel times and UAV operation times within the same framework. To address this gap, this study proposes a spatiotemporal uncertainty-aware task planning framework for vehicle-UAV cooperative remote sensing verification in complex terrain. The main contributions are summarized as follows.

(1) As the core contribution, an Uncertainty-Aware Two-Stage Scheduling Framework (UATSF) is established. It integrates task region generation, collaborative scheduling, and uncertainty assessment within a unified framework. It considers uncertainty in actual vehicle travel times and UAV operation times through a two-stage process: deterministic scheduling optimization is first performed, and Monte Carlo simulation is then used to assess schedule reliability under uncertain operating conditions.
(2) Within the UATSF, a Capability-Aware Task Region Generation (CTRG) method is proposed. It groups scattered parcels into executable task regions under UAV endurance and communication constraints. A region merging strategy is used to reduce task fragmentation and unnecessary vehicle relocation.
(3) A Dual-Channel Genetic Algorithm (DC-GA) is developed as the scheduling component of the UATSF. It jointly optimizes vehicle assignment and region visitation order. An embedded local search mechanism is used to further improve the scheduling solution.
(4) Extensive experiments are conducted in two real-world natural resource monitoring areas in Hunan Province, China. The results demonstrate that the proposed framework can effectively improve task planning efficiency while maintaining schedule reliability.

The remainder of this paper is organized as follows. Section 2 reviews related work. Section 3 presents the proposed method. Section 4 reports the experimental results. Section 5 discusses the limitations of this study and outlines future work. Section 6 concludes the paper.

## 2. Related Work

Vehicle routing and UAV path planning provide the basis for vehicle-UAV cooperative operations. Conventional vehicle routing studies mainly optimize task assignment and visitation order under road network and vehicle capacity constraints. Yao et al. [16] combined Simulated Annealing (SA) with the Sparrow Search Algorithm (SSA). The proposed SA-SSA algorithm generated high-quality routes for large urban logistics networks. However, the method only considered ground vehicles. UAV operations and air-ground synchronization were not included. UAV path planning mainly focuses on waypoint navigation, target visitation, and area coverage. Fu et al. [17] developed a remote sensing image-driven coverage planning method based on deep reinforcement learning. Environmental information was used to guide UAV movement in complex agricultural areas. The method improved coverage efficiency and path quality. However, the UAV operated independently. Vehicle support, battery charging, and fleet-level task assignment were outside the planning model.

Vehicle-UAV cooperative path planning jointly considers ground movement and aerial operations. Xu et al. [18] studied cooperative coverage using Unmanned Ground Vehicles (UGVs) and UAVs. Their model considered static obstacles and no-fly zones. A three-stage alternating optimization algorithm was proposed to optimize the coupled paths. The method improved coverage efficiency in urban environments but assumed deterministic operating conditions. Its coverage targets also differed from scattered and irregular verification parcels. Long et al. [19] developed a vehicle-UAV cooperative framework for image-based three-dimensional reconstruction. The framework jointly optimized task-region partitioning and computation offloading, thereby connecting UAV data acquisition with subsequent processing. Although the method reduced operation time in large-scale scenes, it mainly considered continuous and regular reconstruction areas. Parcel-level coverage and multi-vehicle/multi-UAV scheduling were not addressed. Related truck-UAV routing studies have also considered operational uncertainty. Yin et al. [20] developed a budgeted robust routing model for humanitarian logistics under uncertain demand and truck travel times. However, the tasks were represented by affected-area nodes, and UAV travel time was deterministic. Meng et al. [21] studied multi-visit UAV-assisted routing with stochastic truck travel times and soft time windows. Their two-stage model determined truck routes and UAV flights in the first stage and optimized truck waiting times in the second stage. A rolling-horizon strategy was used to update waiting-time decisions during execution, while UAV flight time remained deterministic. Wang et al. [22] considered uncertain UAV speeds in cooperative delivery and pickup. They used K-means clustering to determine truck stops and developed a data-driven robust model to jointly optimize the truck route and UAV schedule. However, truck speed was fixed, and the tasks were still represented by customer nodes. In the context of natural resource surveys, our previous LandExplorer system [12] demonstrated the feasibility of vehicle-UAV cooperative sensing. The vehicle provided transportation, charging, and communication support, while the UAV acquired remote sensing data. However, its planning function mainly focused on local UAV coverage paths. It did not jointly optimize task-region assignment and visitation order for multiple vehicle-UAV pairs.

Table 1 summarizes the main differences among these studies in terms of application, objective, cooperative setting, and uncertainty. Existing studies mainly focus on logistics, urban coverage, or three-dimensional reconstruction. Their tasks are generally represented by customer nodes, coverage targets, or regular regions rather than irregular remote sensing parcels. Moreover, most methods consider either ground or aerial uncertainty, while the joint effects of vehicle and UAV operation-time uncertainty remain insufficiently studied. An integrated method for task-region generation and multi-vehicle/multi-UAV scheduling is, therefore, still needed for natural resource verification.

## 3. Method

### 3.1. Method Overview

**Figure 4:**
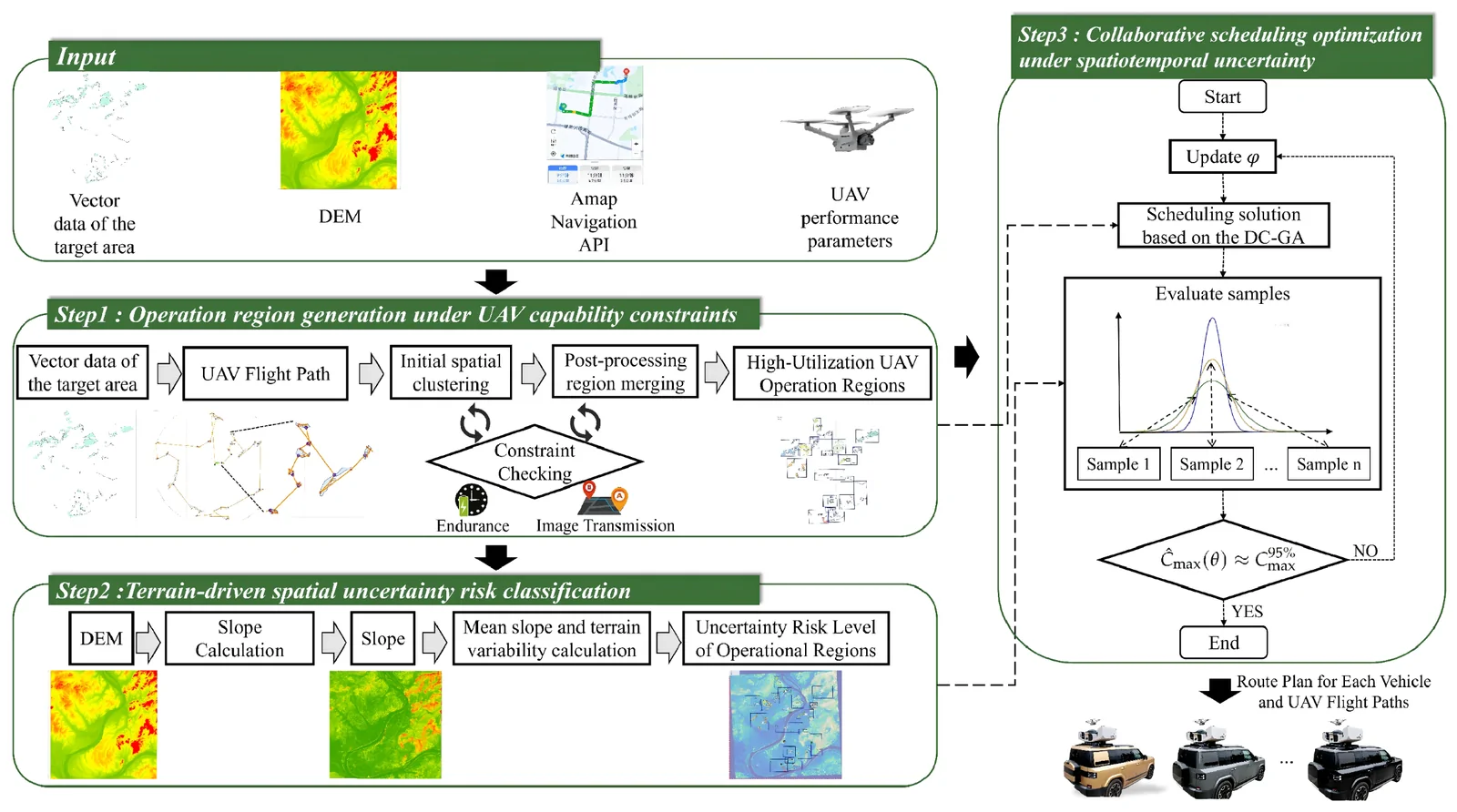
The overall framework (the Chinese labels in the map screenshot are place names displayed by AMap, a Chinese digital mapping and navigation application).

To address the challenges of terrain constraints, limited UAV endurance, and spatiotemporal uncertainty in remote sensing verification tasks over complex terrain, this paper proposes a vehicle–UAV collaborative task planning method that explicitly considers spatiotemporal uncertainty. The proposed method is built upon the collaborative operation of a ground vehicle and a UAV. This framework enables efficient and reliable execution of remote sensing verification tasks in complex environments. The overall framework is illustrated in Figure 4.

- Task region generation under UAV capability constraints. The candidate verification parcels are typically scattered and vary considerably in size. An initial spatial clustering procedure is first performed according to the UAV flight endurance and communication range constraints. This procedure generates a set of basic task regions that can be completed independently by a single UAV. The resulting regions are then further refined by considering their spatial adjacency and the relocation cost of the ground vehicle. Neighboring small regions are merged when appropriate to reduce region fragmentation and avoid frequent vehicle relocations. This two-stage strategy, consisting of initial clustering and region merging, satisfies UAV operational constraints while improving the efficiency of vehicle–UAV collaborative scheduling.
- Terrain-driven spatial uncertainty risk classification. Complex terrain can significantly affect UAV flight stability and operational efficiency. Therefore, a terrain complexity metric is constructed using digital elevation model (DEM) data. A slope raster is first generated from the DEM. The mean and standard deviation of the slope within each task region are then calculated to characterize both the overall terrain gradient and local terrain variability. Based on these features, a terrain complexity metric is established. Task regions are subsequently classified into low-, medium-, and high-risk categories according to the quantiles of terrain complexity. Each risk category is associated with a different level of stochastic disturbance to characterize the spatial heterogeneity of operational uncertainty in complex terrain.
- Deterministic collaborative scheduling and stochastic robustness assessment. Uncertainty during task execution is modeled from both temporal and spatial perspectives. On the temporal side, vehicle travel time is obtained from the road network using the Amap API [23], which accounts for road conditions, traffic status, and route differences. The UAV operation time within each region is modeled using an uncertainty factor to capture the effects of wind disturbances, GPS positioning errors, and complex terrain on flight efficiency. On the spatial side, different uncertainty distribution parameters are assigned to task regions according to their risk categories. This design reflects the spatial variation of operational uncertainty across complex terrain. Based on these models, a vehicle–UAV collaborative task planning model is formulated with the objective of minimizing the overall mission completion time. An improved genetic algorithm is then developed to jointly optimize task allocation and region visiting sequences. The operation time of UAVs in complex environments is inherently stochastic. Therefore, deterministic scheduling results alone cannot adequately reflect the reliability of a planning scheme. To address this issue, Monte Carlo simulation is employed to evaluate the robustness of the optimized solution. During the simulation, uncertainty distribution parameters are assigned according to the risk category of each task region. The corresponding operation time is then randomly sampled to simulate task execution under uncertain conditions. A large number of simulation runs are performed, and high-quantile performance metrics are analyzed to comprehensively evaluate the stability and robustness of the scheduling strategy.

The proposed framework integrates terrain information derived from remote sensing with spatiotemporal uncertainty modeling into the vehicle–UAV collaborative planning process. As a result, it supplements deterministic collaborative scheduling with a risk-aware stochastic robustness assessment.

**Figure 5:**
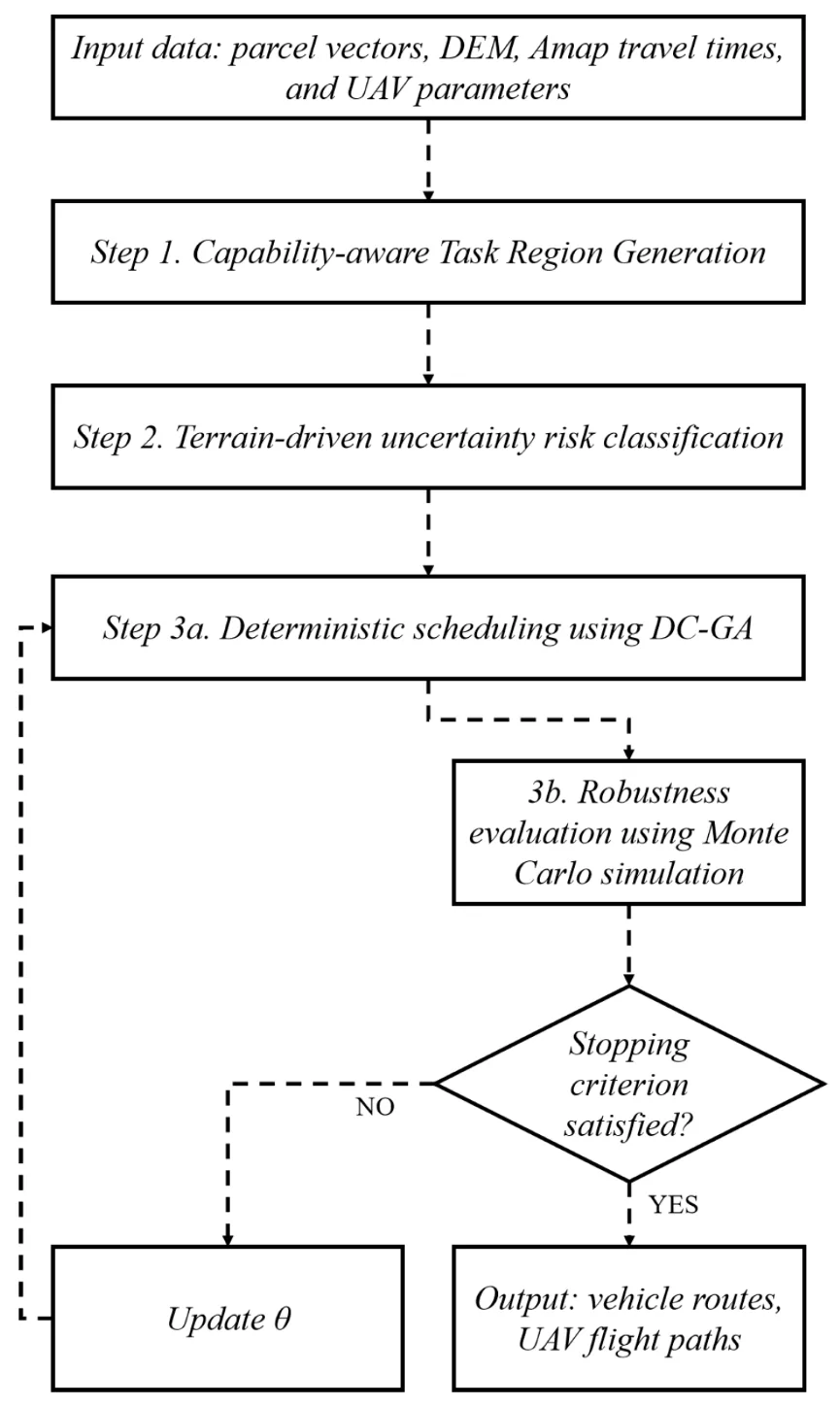
Simplified workflow and interactions among the main components of the proposed framework.

To further clarify the interaction and execution sequence among these components, Figure 5 presents a simplified workflow of the proposed framework. The CTRG method first generates feasible task regions according to the parcel data and UAV capability constraints. Terrain information derived from the DEM is then used to assign an uncertainty risk level to each region. The generated regions and risk information, together with the road-network travel times, are passed to the UATSF. Within the UATSF, the DC-GA generates a deterministic scheduling solution, which is subsequently evaluated through Monte Carlo simulation. If the stopping criterion is not satisfied, θ is updated and the scheduling process is repeated; otherwise, the final vehicle routes, UAV flight paths, and scheduling reliability results are returned.

### 3.2. Capability-Aware Task Region Generation

The candidate verification parcels are typically characterized by scattered spatial distributions, large quantities, and substantial variations in size [24,25]. Directly treating each parcel as an individual scheduling task would dramatically increase the scheduling scale. It would also result in frequent vehicle stops and repeated UAV takeoffs and landings, leading to excessive non-operational time and reduced inspection efficiency. Therefore, before vehicle–UAV collaborative scheduling, the original parcels should first be organized into task regions. Multiple spatially adjacent parcels that can be completed within a single UAV flight are grouped into one task region. This strategy reduces the scheduling scale and improves the efficiency of subsequent path planning and task allocation.

To address this problem, this paper proposes the CTRG method. The proposed method jointly considers the spatial distribution of verification parcels and the operational capability of the UAV. It ensures that each task region can be completed within a single UAV mission while maximizing spatial compactness and minimizing the number of task regions. The generated task regions provide high-quality task units for subsequent vehicle–UAV collaborative scheduling.

#### 3.2.1. Parcel Operation Cost Modeling and Constraint-Driven Clustering

Let $P=\left\{p_{1},p_{2},\ldots ,p_{N}\right\}$ denote the set of candidate verification parcels within the study area. To ensure that the clustering process accurately reflects the actual operational workload, the operation cost of each parcel is first modeled. For parcel $p_{i}$, its geometric center is denoted by $c_{i}$.

Remote sensing verification requires comprehensive image acquisition for each parcel. In practice, the UAV typically flies along the parcel boundary and hovers at predefined locations to capture images, thereby recording the parcel conditions as completely as possible. To approximate the actual inspection trajectory, an ordered sequence of image acquisition points is generated by uniformly sampling the parcel boundary with a fixed sampling interval δ. The resulting sequence is denoted by $Q_{i}=\left\{q_{i1},\ldots ,q_{im_{i}}\right\}$, where $m_{i}$ is the number of image acquisition points. The last point satisfies $q_{im_{i}}=q_{i1}$, forming a closed inspection loop.

The operation cost of parcel $p_{i}$, denoted by $t_{i}$, consists of the boundary coverage flight time and the image acquisition time. It is computed as follows:

(1) $$L_{i}=\sum_{k=1}^{m_{i}-1}\left||q_{i,k+1}-q_{i,k}\right|{|}_{2},$$

(2) $$t_{i}=\frac{L_{i}}{v_{u}}+m_{i}\tau_{c},$$

where $L_{i}$ denotes the boundary coverage flight path of parcel $p_{i}$, $v_{u}$ is the UAV flight speed, and $\tau_{c}$ represents the maneuvering time required for a single image acquisition.

The two terms in $t_{i}$ quantify different sources of workload: $L_{i}/v_{u}$ is the time required to traverse the parcel boundary, whereas $m_{i}\tau_{c}$ is the cumulative time spent maneuvering and acquiring images.

Based on the operation cost model, all candidate verification parcels are partitioned through constraint-driven spatial clustering. Let *K* denote the current number of clusters. The cluster centroids are initialized using the K-means++ strategy. During the assignment step, only spatial proximity is considered. Specifically, each parcel $p_{i}$ is assigned to the region with the nearest centroid:

(3) $$R_{k}=\arg\min_{j\in \left\{1,\ldots ,K\right\}}\left|\right|c_{i}-\mu_{j}{\left|\right|}_{2}.$$

After all parcels have been assigned, the centroid of each region is updated as the arithmetic mean of the parcel centers within that region:

(4) $$\mu_{k}=\frac{1}{|R_{k}|}\sum_{p_{i}\in R_{k}}c_{i}.$$

The assignment and centroid update steps are repeated iteratively until the maximum centroid displacement falls below a predefined convergence threshold. The resulting partition under the current cluster number *K* is denoted by R(K).

After spatial clustering, each task region must be verified to ensure that it satisfies the UAV operational constraints. Since both the parcel visiting sequence and the ordering of image acquisition points directly affect the flight distance and operation time, the operation time of each region is estimated using the following procedure. (i) Parcel visiting sequence. The centroid of region $R_{k}$, denoted by $\mu_{k}$, is taken as the UAV takeoff and landing location. A nearest-neighbor heuristic is adopted to determine the parcel visiting sequence. The UAV starts from the parcel closest to $\mu_{k}$. At each step, the nearest unvisited parcel is selected until all parcels in the region have been visited. (ii) Waypoint ordering within each parcel. For each parcel, the image acquisition points are sorted according to their polar angles with respect to the parcel center. Both clockwise and counterclockwise traversal paths are evaluated, and the shorter one is selected as the boundary coverage path. (iii) Waypoint connection between adjacent parcels. The transition between two consecutive parcels is determined at the waypoint level rather than the parcel-center level. Specifically, let $q_{i}^{\mathrm{exit}}$ denote the last image acquisition point of parcel $p_{i}$. For the subsequent parcel $p_{j}$, the entry point $q_{j}^{\mathrm{entry}}$ is selected as the image acquisition point closest to $q_{i}^{\mathrm{exit}}$.

Based on the above procedure, the total operation time of region $R_{k}$ is computed as follows:

(5) $${\hat{T}}_{k}=\sum_{p_{i}\in R_{k}}t_{i}+\frac{1}{v_{u}}\left(\left|\right|q_{1}^{\mathrm{entry}}-\mu_{k}{\left|\right|}_{2}+\sum_{r=1}^{|R_{k}|-1}\left|\right|q_{r+1}^{\mathrm{entry}}-q_{r}^{\mathrm{exit}}{\left|\right|}_{2}+\left|\right|\mu_{k}-q_{|R_{k}|}^{\mathrm{exit}}{\left|\right|}_{2}\right)+2\tau_{l},$$

where $|R_{k}|$ denotes the number of parcels in region $R_{k}$, and $\tau_{l}$ represents the UAV takeoff or landing time.

Two hard constraints are then evaluated for each task region.

- Flight endurance constraint
  (6) $${\hat{T}}_{k}\leq T_{\max},$$
  where $T_{\max}$ denotes the maximum UAV flight endurance.
- Communication constraint
  (7) $$\max_{q\in Q(R_{k})}||q-\mu_{k}{\left|\right|}_{2}\leq D_{\max},$$
  where $Q(R_{k})$ denotes the set of all image acquisition points within region $R_{k}$, and $D_{\max}$ is the maximum reliable communication range of the UAV.

The endurance constraint limits the total duration of a single UAV flight mission, whereas the communication constraint limits the farthest waypoint that must remain connected to the UAV takeoff and landing location.

If all task regions satisfy both constraints under the current cluster number *K*, the clustering process terminates. Otherwise, the number of clusters is incremented (K←K+1), and the clustering procedure is repeated using the updated value of *K*. This constraint-driven process continues until all task regions satisfy the above constraints. Consequently, the generated task regions are guaranteed to be physically feasible with respect to the UAV operational capabilities.

#### 3.2.2. Post-Processing Region Merging

The initial constraint-driven clustering is designed to ensure that each task region can be completed within a single UAV mission. As a result, it tends to generate a relatively large number of fragmented regions with light workloads. Maintaining these neighboring light-load regions as independent tasks forces the ground vehicle to relocate frequently, leading to increased travel time and additional stop-and-start overhead. To address this issue, a safety-margin-based post-processing merging stage is introduced after the initial clustering. Under the premise that all UAV operational constraints remain satisfied, spatially adjacent regions with compatible workloads are iteratively merged. This strategy reduces the total number of task regions and improves the efficiency of vehicle–UAV collaborative operations.

(1) UAV utilization evaluation and low-utilization region selection

For each task region $R_{k}$ obtained from the initial constraint-driven clustering, let ${\hat{T}}_{k}$ denote its estimated operation time. The UAV utilization of region $R_{k}$, denoted by $l_{k}$, is defined as follows:

(8) $$l_{k}=\frac{{\hat{T}}_{k}}{T_{\max}},$$

where $T_{\max}$ is the maximum UAV flight endurance. If $l_{k}<\eta$, the corresponding task region is identified as a low-utilization region and added to the candidate set for region merging. In this study, the threshold is set to η=0.8.

The threshold η=0.8 identifies regions that use less than 80% of the available flight endurance. Thus, at least 20% of the endurance remains available, leaving room for possible merging with a nearby region. This value is used only for initial candidate selection; every merged region must still pass the subsequent endurance and communication checks.

(2) Spatial proximity filtering

To preserve the spatial compactness of task regions, only spatially adjacent low-utilization regions are considered for merging. Let $d_{ij}$ denote the Euclidean distance between the centroids of two task regions $R_{i}$ and $R_{j}$:

(9) $$d_{ij}=\left|\right|\mu_{i}-\mu_{j}{\left|\right|}_{2}.$$

The spatial proximity criterion for region merging is defined as follows:

(10) $$d_{ij}\leq 2D_{\max},$$

where $D_{\max}$ is the maximum reliable image transmission range of the UAV. If the distance between two region centroids exceeds twice the transmission range, the regions are regarded as spatially separated. Merging them would make it difficult for a single takeoff and landing point to cover all target parcels effectively. Such a merge is, therefore, likely to violate the communication constraint and is excluded from further consideration.

(3) Conservative operation time estimation

For each candidate region pair $R_{i}$ and $R_{j}$ that satisfies the spatial proximity criterion, a conservative estimation is first performed to assess the feasibility of merging. This step eliminates clearly infeasible candidates before detailed evaluation, thereby reducing unnecessary computation. Let the merged task region be $R_{ij}=R_{i}\cup R_{j}$. Its conservative estimated operation time, denoted by ${\hat{T}}_{ij}$, is calculated as follows:

(11) $${\hat{T}}_{ij}=\sum_{p\in R_{ij}}t_{p}+\frac{2d_{\max}}{v_{u}}+2\tau_{l},$$

where $d_{\max}$ is the distance from the centroid of the merged region to its farthest parcel centroid. The merged region is accepted for further evaluation only if

(12) $${\hat{T}}_{ij}\leq \rho T_{\max},$$

where ρ is set to 0.95. If the conservative estimate exceeds 95% of the maximum UAV flight endurance, the candidate pair is discarded directly. Otherwise, it proceeds to the subsequent stage for detailed feasibility verification.

In this estimate, $2d_{\max}/v_{u}$ approximates the outward-and-return flight time between the new region center and its most distant parcel. The factor ρ=0.95 reserves a 5% endurance margin to account for differences between this preliminary estimate and the exact route. It is used only for initial screening; every accepted merge must still pass the detailed feasibility verification.

(4) Precise feasibility verification after region merging

For each candidate pair that passes the conservative estimation, the merging operation is performed by transferring all patches in $R_{j}$ to $R_{i}$. The centroid of the merged region is then updated, and $R_{j}$ is removed from the region set. Subsequently, the UAV flight path generation procedure described in Section 3.2.1 is applied to the merged region. The operation time, denoted by ${\hat{T}}_{ij}^{\mathrm{new}}$, is recalculated according to the actual spatial distribution of all parcels within the merged region. The merged region is accepted only if it satisfies both hard constraints defined in Section 3.2.1, namely, the flight endurance constraint and the communication range constraint.

If both constraints are satisfied, the merging operation is retained. Otherwise, the merge is rolled back immediately, and the original partition of $R_{i}$ and $R_{j}$ is restored. This trial-and-verification strategy guarantees monotonic feasibility throughout the merging process. Every accepted merge preserves the feasibility of the current solution. Through the above iterative merging procedure, the proposed CTRG method further aggregates fragmented low-utilization regions after the initial constraint-driven clustering. The resulting task regions exhibit higher UAV utilization, improved spatial compactness, and a more balanced workload distribution. These refined task regions provide more efficient inputs for the subsequent vehicle–UAV collaborative task scheduling.

### 3.3. Terrain-Driven Spatial Uncertainty Risk Classification

Complex terrain is one of the primary factors affecting low-altitude UAV flight safety and operational efficiency. Terrain slope, as a fundamental descriptor of surface morphology, characterizes the overall steepness and local variation of the flight environment. Steep and highly variable terrain can alter near-surface airflow, reduce satellite visibility in terrain-obstructed areas, and require more frequent altitude or attitude adjustments. These mechanisms provide an operational link between terrain morphology and fluctuations in UAV operation time. Terrain complexity is, therefore, used as an observable proxy for the relative level of time uncertainty among task regions.

To capture this spatial heterogeneity, the terrain complexity of each task region is quantified and used to classify spatial uncertainty risk levels. These risk levels provide the basis for assigning region-specific uncertainty parameters in the subsequent spatiotemporal scheduling model.

A slope raster is first generated from the DEM of the study area. For each task region $R_{k}$, the Minimum Bounding Rectangle (MBR) is computed, and all slope pixels within the MBR are extracted. Let the set of valid slope values in region $R_{k}$ be the following:

(13) $$S_{k}=\left\{s_{1},s_{2},\ldots ,s_{n}\right\}.$$

The mean slope and slope standard deviation of region $R_{k}$ are calculated as follows:

(14) $${\mathrm{slope}}_{\mu_{k}}=\frac{1}{n}\sum_{i=1}^{n}s_{i},$$

(15) $${\mathrm{slope}}_{\sigma_{k}}=\sqrt{\frac{1}{n-1}\sum_{i=1}^{n}{\left(s_{i}-{\mathrm{slope}}_{\mu_{k}}\right)}^{2}}.$$

The mean slope, ${\mathrm{slope}}_{\mu_{k}}$, characterizes the overall terrain steepness of the region. The slope standard deviation, ${\mathrm{slope}}_{\sigma_{k}}$, measures terrain variability. Based on these two statistics, the terrain complexity of region $R_{k}$ is defined as follows:

(16) $$TC_{k}=0.5\cdot {\mathrm{slope}}_{\mu_{k}}+0.5\cdot {\mathrm{slope}}_{\sigma_{k}}.$$

This metric jointly considers terrain steepness and terrain variability. Equal weights are adopted to balance their contributions, preventing the complexity measure from being dominated by either extreme slope values or terrain heterogeneity. The mean slope represents the overall terrain steepness, whereas the slope standard deviation represents local terrain variation. These two quantities have the same physical unit and describe complementary characteristics of terrain morphology. Assigning equal weights avoids introducing an unsupported preference for either characteristic and provides a transparent and reproducible measure of terrain complexity. The 0.5/0.5 weights are, therefore, adopted as an engineering modeling setting for the present scheduling analysis and can be further refined using field-flight data. After the terrain complexity of all task regions has been obtained, spatial uncertainty risk levels are determined according to the complexity distribution. Let

(17) $$TC=\left\{TC_{1},TC_{2},\ldots ,TC_{M}\right\}$$

denote the set of terrain complexity values for all task regions. The 33.3% and 66.7% percentiles are used as the classification thresholds:

(18) $$Q_{1}=Q_{33.3\%}\left(TC\right),$$

(19) $$Q_{2}=Q_{66.7\%}\left(TC\right).$$

The spatial uncertainty risk level of region $R_{k}$ is then defined as follows:

(20) $$\mathrm{Risk}\left(R_{k}\right)=\left\{\begin{array}{cc} \mathrm{Low}, & TC_{k}\leq Q_{1},\\ \mathrm{Medium}, & Q_{1}<TC_{k}\leq Q_{2},\\ \mathrm{High}, & TC_{k}>Q_{2}. \end{array}\right.$$

Because terrain ranges can differ considerably across landscapes, fixed absolute thresholds may not provide consistent differentiation among task regions in different study areas. Computing $Q_{1}$ and $Q_{2}$ from the terrain-complexity distribution makes the classification data-adaptive and provides three distinguishable risk levels for assigning uncertainty parameters. This design is consistent with the purpose of the model, which is to prioritize relative operation-time uncertainty within a given mission rather than to determine absolute flight-safety levels. Therefore, the quantile thresholds are used as a mission-oriented modeling setting. With the accumulation of field-flight data from different terrains and UAV platforms, transferable absolute thresholds can be further calibrated.

Different risk levels are associated with different uncertainty distribution parameters for UAV operation time. According to the flight characteristics of UAVs in complex terrain, the operation time of each task region is modeled using a truncated normal distribution:

(21) $${\tilde{T}}_{k}∼\mathrm{TruncNorm}\left(\mu ={\hat{T}}_{k},\;\sigma =\sigma_{\mathrm{risk}},\;a=0.9{\hat{T}}_{k},\;b=1.25{\hat{T}}_{k}\right),$$

where ${\hat{T}}_{k}$ is the deterministic estimate of the operation time for task region $R_{k}$, $\sigma_{\mathrm{risk}}$ is the standard deviation determined by the corresponding risk level, and *a* and *b* denote the lower and upper truncation bounds, respectively. Specifically, a smaller standard deviation is assigned to low-risk regions, reflecting the limited variability of operation time over relatively flat terrain. Medium-risk regions are assigned a moderate standard deviation to capture the combined effects of wind disturbances, GPS positioning errors, and other factors in moderately undulating terrain. High-risk regions are associated with a larger standard deviation, representing the substantial variability introduced by strong airflow disturbances, frequent flight control adjustments, and other factors in steep terrain.

In the simulations, the relative standard-deviation coefficient is set to 0.050, 0.125, and 0.250 for low-, medium-, and high-risk regions, respectively. Therefore, the variability assigned to each region is proportional to its deterministic operation time and increases with the terrain-derived risk level.

The truncated normal distribution is used because UAV operation time is expected to fluctuate around the deterministic estimate but remains bounded by practical operating conditions. Centering the distribution at ${\hat{T}}_{k}$ preserves the deterministic estimate as the nominal operation time, while the risk-dependent $\sigma_{\mathrm{risk}}$ controls the amount of variation. The interval from $0.9{\hat{T}}_{k}$ to $1.25{\hat{T}}_{k}$ excludes unrealistically short or long samples, and its wider upper side reflects that operational delays are usually larger than possible time savings. This setting provides controlled stochastic scenarios for comparing scheduling solutions under different terrain-risk levels. For the present experiments, the distribution form, risk-dependent standard deviations, and truncation bounds are treated as consistent engineering modeling assumptions rather than universal constants. Future work will use field-flight records to calibrate these parameters for different UAV platforms and operating environments.

### 3.4. Spatiotemporal Uncertainty-Aware Task Planning for Remote Sensing Monitoring and Verification

After the generation of task regions and terrain-driven uncertainty risk classification, vehicle–UAV cooperative scheduling optimization is further performed by explicitly considering both temporal and spatial uncertainties. Unlike conventional deterministic scheduling, the proposed method models uncertainty from both the temporal and spatial perspectives during task execution.

From the temporal perspective, the travel time between task regions is obtained through the Amap API based on the actual road network, which comprehensively accounts for factors such as road conditions, traffic status, and route differences. Meanwhile, the UAV operation time within each region is modeled using the risk-aware stochastic model described in Section 3.3 to capture the random effects of wind disturbances, GPS positioning errors, flight control response delays, and other factors on operational efficiency.

From the spatial perspective, different uncertainty distribution parameters are assigned to task regions according to their terrain-derived risk levels. Consequently, regions with higher risk exhibit greater variability in operation time, thereby characterizing the spatial heterogeneity of operational risks under complex terrain conditions.

Based on the above spatiotemporal uncertainty modeling, a vehicle–UAV cooperative scheduling model is established to jointly optimize task allocation and region visiting sequences with the objective of minimizing the system makespan. Since random fluctuations in UAV operation time cannot be adequately reflected by deterministic optimization alone, Monte Carlo simulation is further employed to evaluate the robustness of the obtained scheduling solutions. Specifically, operation times are repeatedly sampled from the probability distribution corresponding to the risk level of each region to generate a large number of execution scenarios. The high-percentile makespan under these stochastic scenarios is then calculated to comprehensively evaluate the stability and robustness of the scheduling solution. Through this framework, deterministic scheduling is supplemented by a stochastic robustness assessment under spatiotemporal uncertainty.

#### 3.4.1. Problem Description and Decision Variables

Let $R=\{R_{1},R_{2},\ldots ,R_{M}\}$ denote the set of task regions generated by the CTRG method, and let $V=\{V_{1},V_{2},\ldots ,V_{N_{v}}\}$ denote the set of vehicles. As illustrated in Figure 3, each vehicle is equipped with a highly automated UAV docking station mounted on its roof. The vehicle serves as both a mobile takeoff-and-landing platform and a data relay station for the UAV. The vehicle–UAV cooperative operation proceeds as follows. The vehicle first travels to the center of an assigned task region, which serves as the UAV takeoff and landing point. The UAV then takes off to perform parcel inspection and image acquisition tasks within the region. After completing the operation, the UAV lands on the rooftop docking station, and the vehicle proceeds to the next assigned region. Accordingly, the scheduling problem involves two coupled decision components: (1) *task allocation*, which determines the assignment of task regions to vehicles, and (2) *visiting sequence optimization*, which determines the order in which each vehicle visits its assigned regions.

In this study, the geometric center of each task region is used as a nominal reference for estimating vehicle travel time and UAV operation time. In our field investigations, parcels requiring verification were commonly located in areas affected by human activities, where road accessibility was generally favorable. Thus, the geometric center provides a practical approximation for pre-mission planning, although the actual vehicle stopping and UAV takeoff location may be adjusted according to local road and site conditions.

Let the binary decision variable $x_{v,k}$ indicate whether task region $R_{k}$ is assigned to vehicle $V_{v}$:

(22) $$x_{v,k}=\left\{\begin{array}{cc} 1, & \mathrm{if}\;\mathrm{task}\;\mathrm{region}\;R_{k}\;\mathrm{is}\;\mathrm{assigned}\;\mathrm{to}\;\mathrm{vehicle}\;V_{v},\\ 0, & \mathrm{otherwise}. \end{array}\right.$$

Similarly, let the binary decision variable $y_{v,i,j}$ indicate whether vehicle $V_{v}$ travels directly from task region $R_{i}$ to task region $R_{j}$:

(23) $$y_{v,i,j}=\left\{\begin{array}{cc} 1, & \mathrm{if}\;\mathrm{vehicle}\;V_{v}\;\mathrm{travels}\;\mathrm{directly}\;\mathrm{from}\;R_{i}\;\mathrm{to}\;R_{j},\\ 0, & \mathrm{otherwise}. \end{array}\right.$$

Each vehicle departs from its initial location, visits a sequence of assigned task regions, and finally returns to its starting location, thereby forming a closed route.

#### 3.4.2. Time Modeling

(1) Vehicle travel time

The travel time between task regions is obtained from the Amap API based on the actual road network. The region centers are used only as routing references. The vehicle travel time is calculated by the Amap API based on the actual road network rather than the Euclidean distance between region centers. Let $T_{i,j}^{\mathrm{travel}}$ denote the travel time from the center of task region $R_{i}$ to that of task region $R_{j}$. This value is computed by the API considering real-time traffic conditions and road network topology. Compared with conventional approaches that estimate travel time using road distance and a predefined constant vehicle speed, the API-based travel time captures practical traffic factors, including road hierarchy, turning restrictions, signal delays, and traffic congestion. Consequently, systematic errors introduced by the constant-speed assumption are effectively reduced, resulting in travel time estimates that more accurately reflect real operational conditions.

(2) UAV operation time

The UAV operation time for task region $R_{k}$ consists of a deterministic baseline component and a stochastic disturbance component. The deterministic baseline operation time, denoted by ${\hat{T}}_{k}$, is calculated using Equation (5) in Section 3.2.1, representing the estimated operation time under ideal conditions. During actual operation, however, the execution time is affected by stochastic factors such as wind disturbances, GPS positioning errors, and flight controller response delays. Consequently, the actual operation time ${\tilde{T}}_{k}$ is modeled as a random variable following a truncated normal distribution associated with the terrain-derived risk level of the task region, as described in Equation (21).

(3) UAV charging waiting time

After completing the operation in one region, the UAV may need to recharge on the vehicle before executing the next task. To ensure sufficient operational safety margins against environmental disturbances, route adjustments, and other uncertainties during flight, it is assumed that the UAV is fully recharged before each new operation.

Let $T_{\max}$ denote the maximum flight endurance of the UAV under a full battery, and let $T_{\mathrm{charge}}$ denote the time required for a full recharge. If the UAV spends ${\tilde{T}}_{i}$ operating in region $R_{i}$, the corresponding battery consumption ratio is given by ${\tilde{T}}_{i}/T_{\max}$. Therefore, the required charging time is

(24) $$T_{i}^{\mathrm{chg}}=\frac{{\tilde{T}}_{i}}{T_{\max}}T_{\mathrm{charge}}.$$

The charging time expression is a scheduling-level linear approximation that assumes battery consumption is proportional to operation time and charging proceeds at a constant average rate. It is used to estimate whether vehicle travel can cover the charging requirement between consecutive tasks rather than to reproduce detailed battery electrochemical and thermal dynamics. Additional flight duration caused by wind disturbances and repeated attitude adjustments is partly reflected by the stochastic operation time ${\tilde{T}}_{i}$.

If the vehicle travel time from region $R_{i}$ to region $R_{j}$, i.e., $T_{i,j}^{\mathrm{travel}}$, is sufficient to complete the required charging process, no additional waiting time is incurred. Otherwise, the vehicle must wait until charging is completed before the UAV can execute the next task. Accordingly, the time interval between completing the operation in region $R_{i}$ and starting the operation in region $R_{j}$ is expressed as

(25) $$W_{i,j}=T_{i,j}^{\mathrm{travel}}+\max\left(0,\frac{{\tilde{T}}_{i}}{T_{\max}}T_{\mathrm{charge}}-T_{i,j}^{\mathrm{travel}}\right).$$

Equation (25) is equivalently $W_{i,j}=\max\left\{T_{i,j}^{\mathrm{travel}},T_{i}^{\mathrm{chg}}\right\}$. This form shows that vehicle travel and UAV charging occur in parallel: additional idle time is incurred only when the required charging time is longer than the travel time to the next region.

#### 3.4.3. Optimization Model

The objective of the proposed scheduling model is to minimize the system makespan, defined as the completion time of the last vehicle returning to its depot. For a given scheduling solution, the completion time of vehicle $V_{v}$ is calculated as

(26) $$C_{v}=\sum_{r=1}^{|R_{v}|}\left(W_{\mathrm{prev},r}+{\tilde{T}}_{r}\right),$$

where $R_{v}$ denotes the sequence of task regions assigned to vehicle $V_{v}$, $W_{\mathrm{prev},r}$ represents the transition time from the previous location (either another task region or the depot) to task region *r*, and ${\tilde{T}}_{r}$ denotes the actual UAV operation time in region *r*.

The system makespan is defined as the maximum completion time among all vehicles:

(27) $$C_{\max}=\max_{v\in V}C_{v}.$$

The maximum operator identifies the latest-finishing vehicle as the bottleneck of the collaborative system. Minimizing $C_{\max}$, therefore, discourages highly unbalanced task assignments, even when their total travel time may be small, and promotes a more balanced use of the available vehicle-UAV pairs.

Since the scheduling structure (i.e., task allocation and visiting sequence) is primarily determined by the expected operation time, while stochastic disturbances mainly affect solution robustness, the deterministic baseline operation time ${\hat{T}}_{r}$ is used in place of the stochastic variable ${\tilde{T}}_{r}$ during optimization. Accordingly, the deterministic scheduling model is formulated as follows:

(28) $$\min{\hat{C}}_{\max}=\min\left\{\max_{v\in V}\sum_{r=1}^{|R_{v}|}\left(W_{\mathrm{prev},r}+{\hat{T}}_{r}\right)\right\}.$$

The optimization is subject to the following constraints:

(29) $$\begin{array}{cccc} \; & \sum_{v\in V}x_{v,k}=1,\; & \; & \forall k\in R, \end{array}$$

(30) $$\begin{array}{cccc} \; & \sum_{i\in R\cup \{0\}}y_{v,i,j}=x_{v,j},\; & \; & \forall v\in V,\;j\in R, \end{array}$$

(31) $$\begin{array}{cccc} \; & \sum_{j\in R\cup \{0\}}y_{v,i,j}=x_{v,i},\; & \; & \forall v\in V,\;i\in R, \end{array}$$

where Constraint (29) ensures that each task region is assigned to exactly one vehicle, while Constraints (30) and (31) guarantee that the visiting sequence of each vehicle forms a closed route beginning and ending at the depot. In addition, the single-region UAV endurance constraint and communication constraint have already been guaranteed during the CTRG-based task region generation process and, therefore, are not explicitly included in the optimization model.

#### 3.4.4. Solution Method

To solve the proposed scheduling problem under spatiotemporal uncertainty, the UATSF is developed, as illustrated in Figure 4. The framework consists of two sequential stages. In the first stage, a deterministic scheduling problem is solved using the baseline operation times to obtain an optimal vehicle–UAV cooperative scheduling solution. In the second stage, the obtained schedule is evaluated through Monte Carlo simulation to quantify its robustness under stochastic operation-time uncertainty.

Therefore, the UATSF should be understood as deterministic optimization followed by stochastic robustness assessment, rather than as a fully stochastic optimization model in which random variables are directly embedded in the objective function.

(1) Dual-channel coded genetic algorithm

The deterministic scheduling problem is solved using the DC-GA specifically designed for the heterogeneous decision variables of task allocation and visiting sequence. Each chromosome is encoded as a real-valued vector:

(32) $$X=[x_{1},x_{2},\ldots ,x_{M}],$$

where *M* is the number of task regions. For each gene $x_{k}$, the integer part,

(33) $$\left\lfloor x_{k}\right\rfloor \in \left\{0,\ldots ,N_{v}-1\right\},$$

indicates the vehicle assigned to task region $R_{k}$, while the fractional part,

(34) $$\mathrm{frac}\left(x_{k}\right)\in \left[0,1\right),$$

represents the visiting priority of region $R_{k}$ within the assigned vehicle route.

During decoding, task regions are first assigned to different vehicles according to the integer parts of the genes. Subsequently, the regions assigned to each vehicle are sorted in ascending order of their fractional values to obtain the complete visiting sequence. Compared with conventional segmented encodings, the proposed dual-channel representation simultaneously encodes task allocation and visiting sequence within a single gene. Consequently, each genetic operation is capable of exploring both decision subspaces simultaneously, thereby improving the search efficiency and reducing the coupling problem between assignment and sequencing.

Although the genetic algorithm provides strong global search capability for discovering promising task allocation structures, its local optimization ability for visiting sequences under fixed assignments is relatively limited. Therefore, a local search operator is embedded into the evolutionary process. Without modifying the vehicle assignment, the operator exchanges two task regions within the same vehicle route and accepts the exchange only if the fitness is improved. The local search is executed immediately after population initialization and after each crossover–mutation operation. The former improves the quality of the initial population, whereas the latter repairs route deterioration introduced during evolution. Consequently, global exploration and local exploitation are effectively combined to improve both solution quality and convergence speed.

(2) Robustness evaluation based on Monte Carlo simulation

The DC-GA determines the optimal scheduling structure based on the deterministic baseline operation times. However, the deterministic objective value alone cannot adequately characterize the schedule performance under stochastic operating conditions. Therefore, an uncertainty parameter θ∈[0,1] is introduced to construct a family of deterministic operation times:

(35) $${\hat{T}}_{k}\left(\theta \right)=\theta {\hat{T}}_{k}^{\min}+\left(1-\theta \right){\hat{T}}_{k}^{\max},$$

where ${\hat{T}}_{k}^{\min}$ and ${\hat{T}}_{k}^{\max}$ denote the lower and upper bounds of the operation time, respectively. When θ=0, the operation time reaches its upper bound, whereas θ=1 corresponds to the lower bound. Thus, increasing θ continuously shifts the deterministic input from a conservative worst-case estimate toward an optimistic best-case estimate.

The parameter is scanned using a step size of τ=0.1:

(36) θ∈{0,0.1,…,1.0}.

For each value of θ, the DC-GA is executed to obtain the corresponding optimal scheduling solution together with its deterministic makespan ${\hat{C}}_{\max}\left(\theta \right)$.

Subsequently, the obtained schedule is evaluated using Monte Carlo simulation. For each task region, $N_{\mathrm{MC}}$ realizations of the operation time are independently sampled from the truncated normal distribution associated with its terrain-derived risk level. Based on the obtained schedule, the system makespan is calculated for all Monte Carlo realizations and sorted in ascending order. The $95^{\mathrm{th}}$ percentile of the makespan distribution is then computed as

(37) $$C_{\max}^{95\%},$$

which represents the completion time that is not exceeded in 95% of all simulated scenarios. This metric provides a conservative estimate of the schedule completion time and serves as a robustness indicator under stochastic operating conditions.

The parameter scanning process terminates when

(38) $${\hat{C}}_{\max}\left(\theta \right)\approx C_{\max}^{95\%}.$$

This stopping criterion indicates that the deterministic operation times generated by the corresponding value of θ approximate the modeled uncertainty level under the assumed simulation settings. Within these settings, the deterministic objective value serves as a surrogate for the simulated robust completion time, enabling the selected scheduling solution to balance optimization performance and robustness.

## 4. Experiments

### 4.1. Study Areas

**Figure 6:**
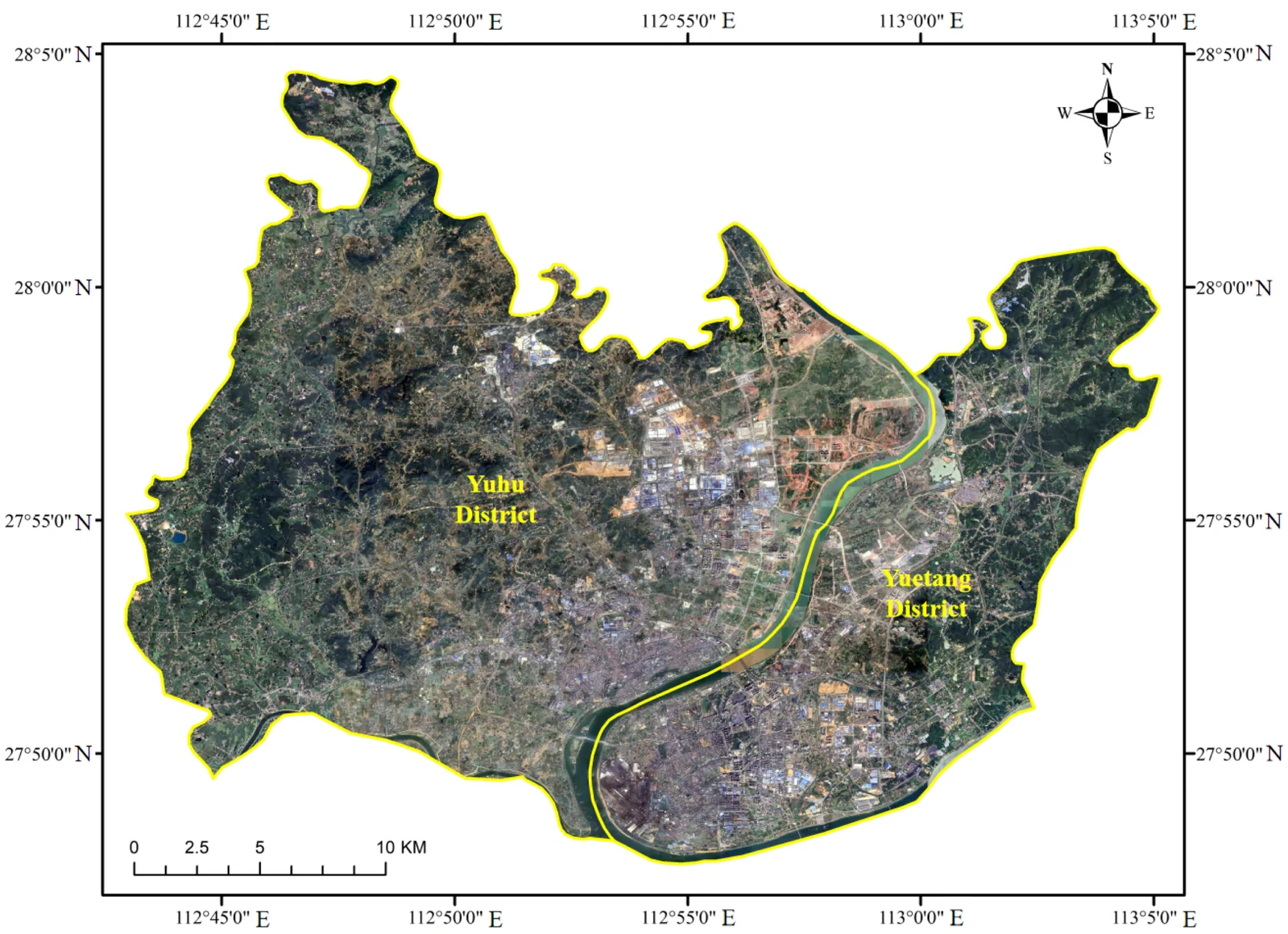
Overview of the Xiangtan Study Area.

**Figure 7:**
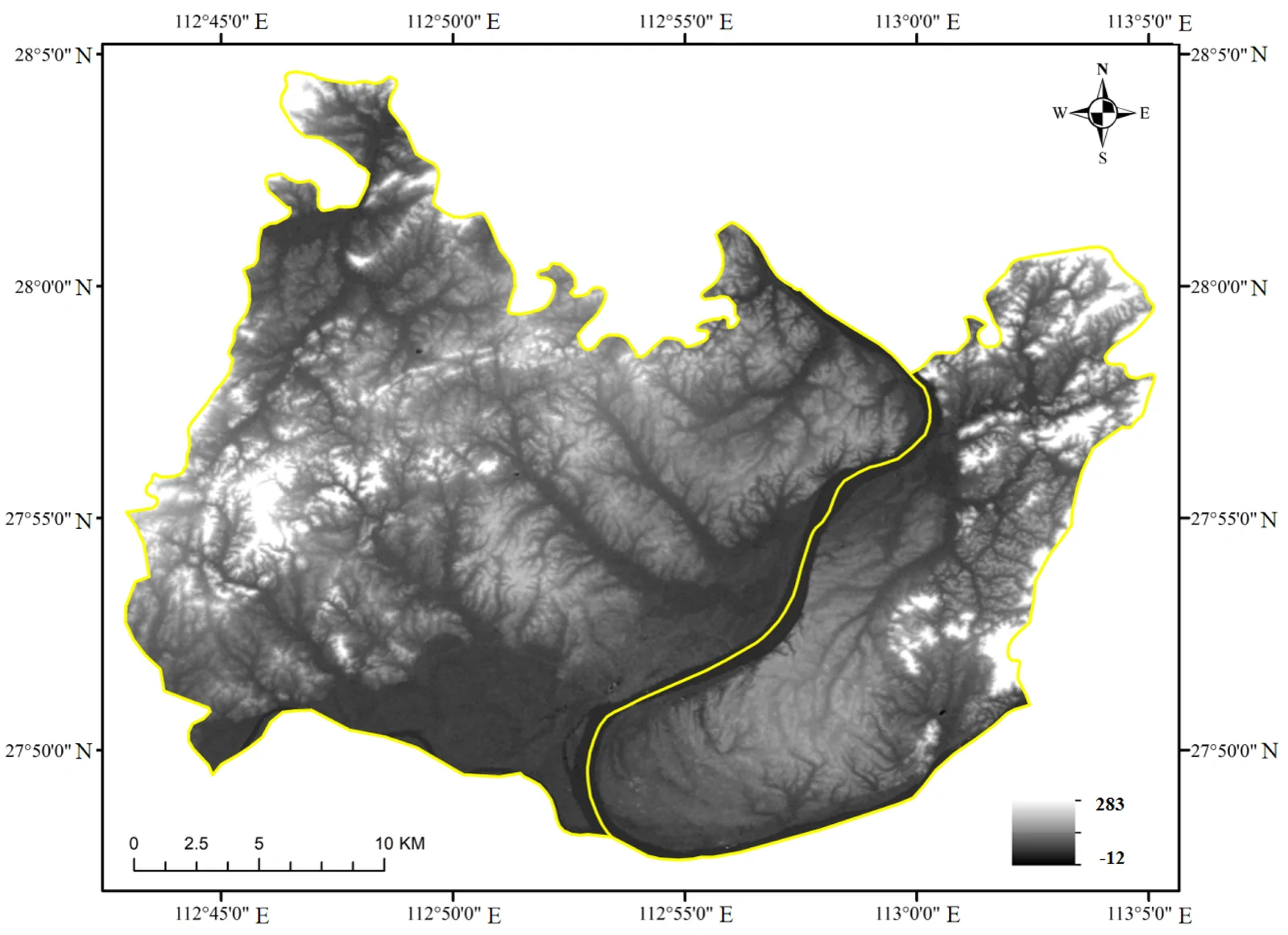
DEM of the Xiangtan Study Area.

**Figure 8:**
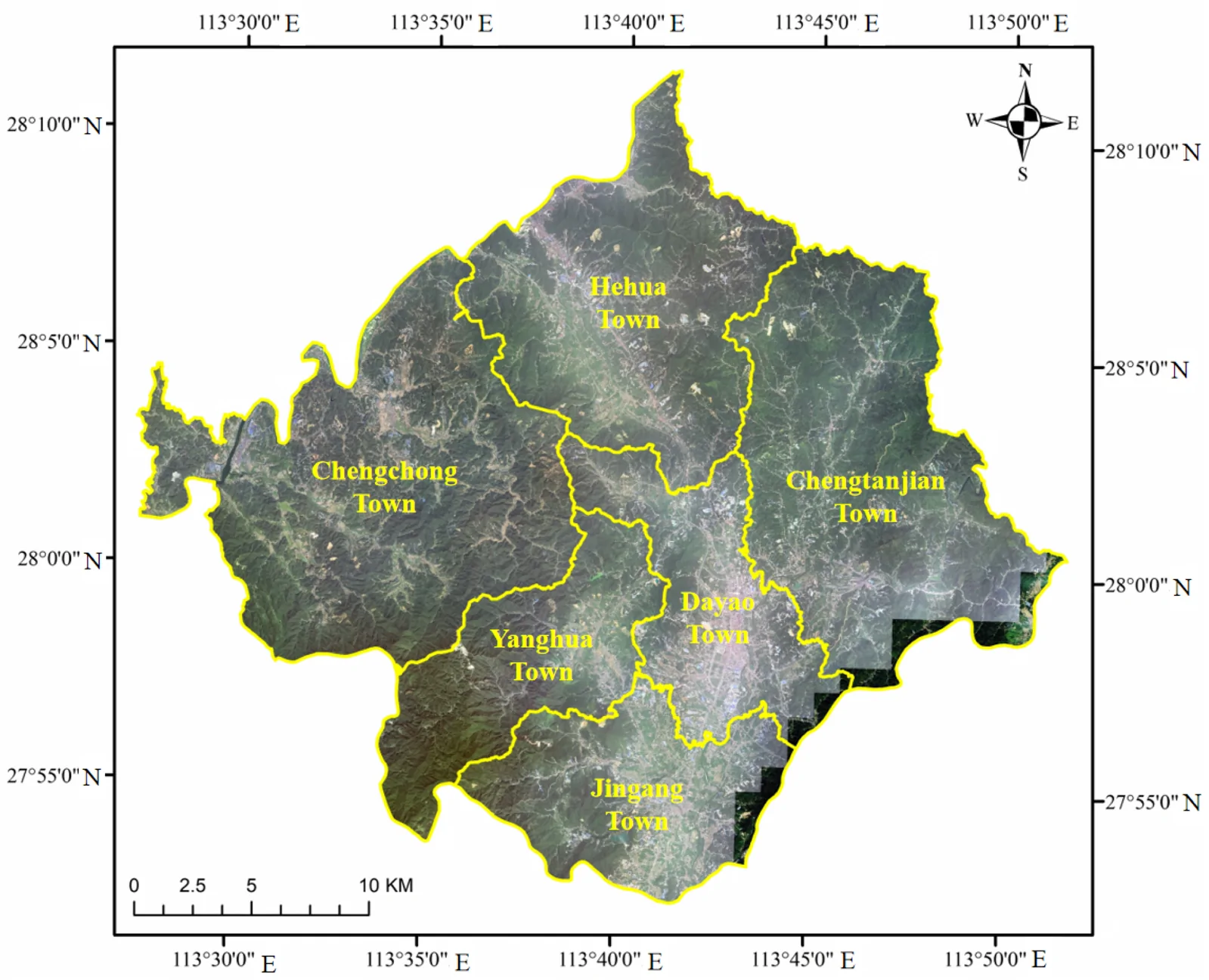
Overview of the Dayao Study Area.

**Figure 9:**
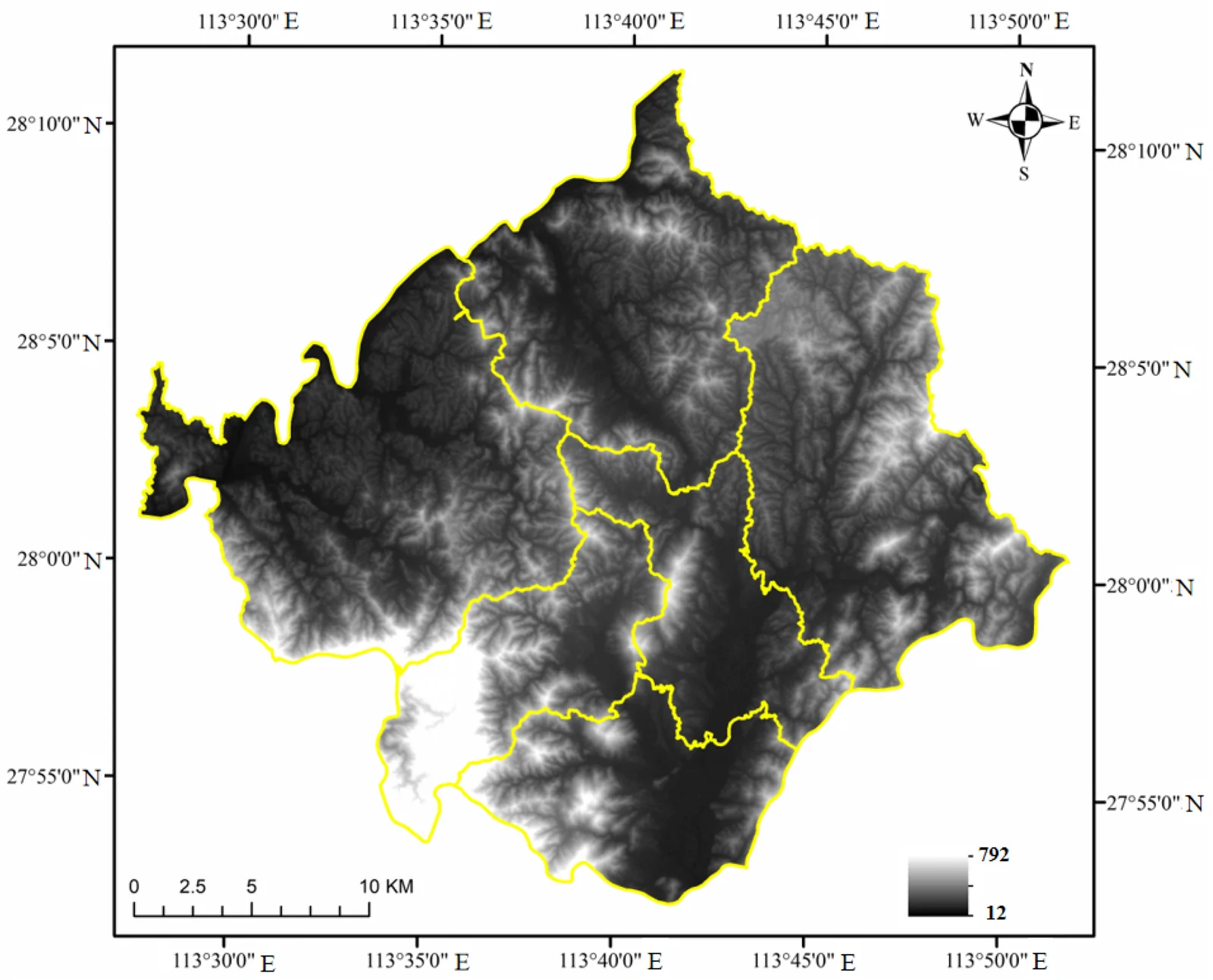
DEM of the Dayao Study Area.

As illustrated in Figure 6, Figure 7, Figure 8 and Figure 9, two study areas were selected to evaluate the proposed method: Xiangtan City and Dayao Town in Liuyang City, Hunan Province, China. The two study areas exhibit distinct characteristics in terms of terrain conditions, parcel size, and spatial distribution, providing representative scenarios for validating the applicability of the proposed method [26,27].

The Xiangtan study area contains 145 candidate verification parcels, while the Dayao study area contains 478 candidate verification parcels. The parcels in the Xiangtan study area are generally larger and have complex, irregular boundaries. In contrast, the parcels in the Dayao study area are typically smaller and more sparsely distributed, exhibiting pronounced spatial fragmentation.

The substantial differences between the two study areas provide a comprehensive basis for evaluating the performance and robustness of the proposed method under diverse operational scenarios.

The UAV-related parameters used in the experiments were set with reference to the typical performance ranges of several commonly used DJI UAVs (SZ DJI Technology Co., Ltd., Shenzhen, China) [28,29]. Specifically, the maximum flight endurance $T_{\max}$ was set to 2400 s (40 min), the maximum reliable image-transmission radius $D_{\max}$ was set to 5000 m, the UAV flight speed $v_{u}$ was set to 15 m/s, and the full charging time $T_{\mathrm{charge}}$ was set to 2400 s (40 min). In addition, image acquisition points were generated along the parcel boundaries at an interval δ of 500 m. The time required for maneuvering and image acquisition at each point, $\tau_{c}$, was set to 2 s, while the time required for either takeoff or landing, $\tau_{l}$, was set to 30 s. These settings provide a practical representation of the operational capabilities of commonly used UAV platforms.

### 4.2. Experimental Results

To evaluate the effectiveness of the proposed methods, comprehensive experiments were conducted on the Xiangtan and Dayao study areas. Specifically, ablation studies were first designed to investigate the contributions of the post-processing region-merging strategy in CTRG and the local search mechanism in DC-GA. Subsequently, the optimization performance of DC-GA was compared with several representative metaheuristic algorithms. Finally, the two-stage procedure of UATSF, comprising deterministic optimization and stochastic robustness assessment, was evaluated under stochastic operating conditions.

#### 4.2.1. Ablation Study

To investigate the effectiveness of the proposed region merging strategy and the embedded local search operator, ablation experiments were conducted on both study areas. In the Xiangtan dataset, experiments were performed with three and five vehicles under three traffic periods. Similarly, two traffic periods were considered in the Dayao dataset for both vehicle configurations. The experimental settings provide diverse traffic conditions and spatial distributions for evaluating the contribution of each module.

**Figure 10:**
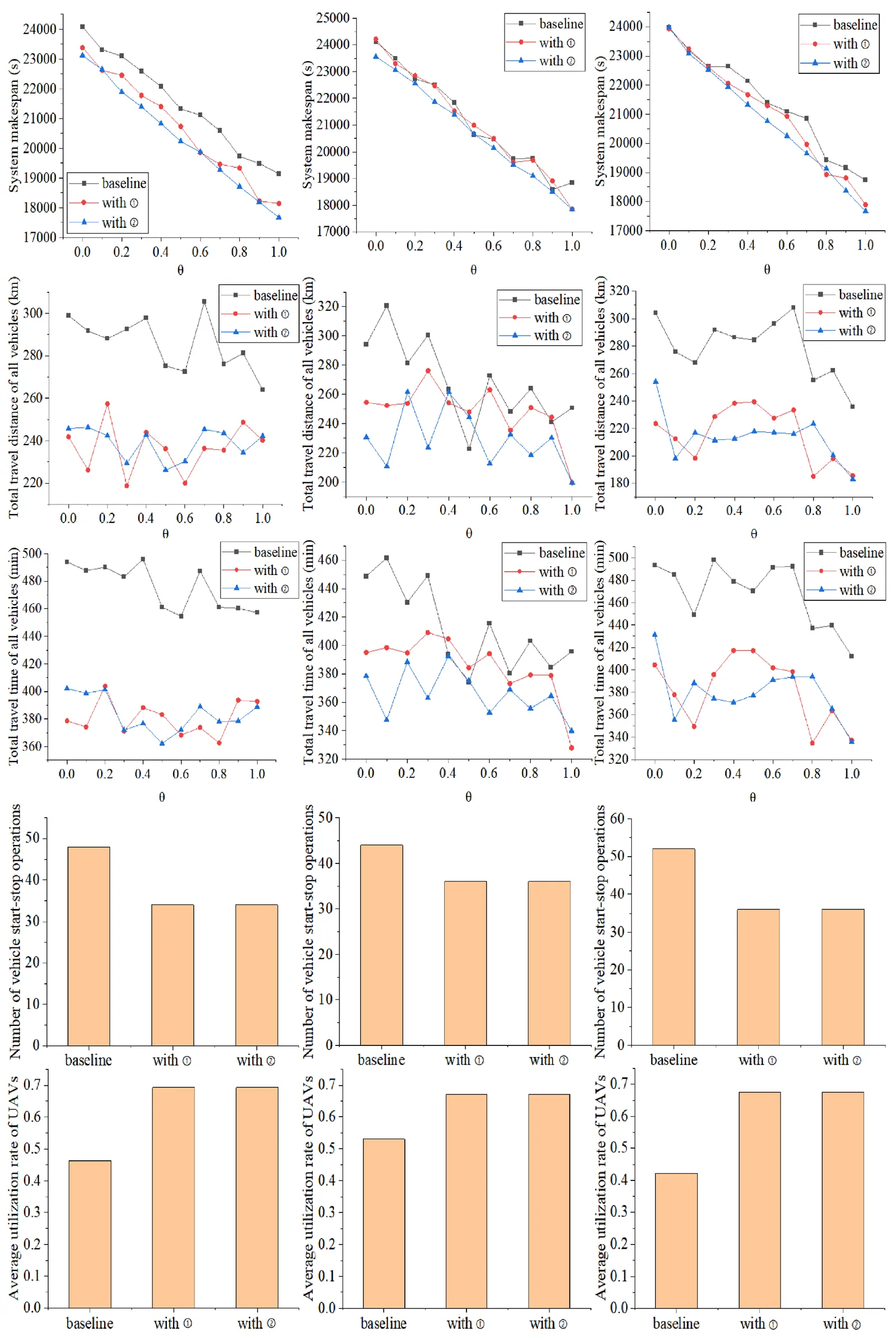
Ablation study results in the Xiangtan study area under the three-vehicle configuration. ① denotes the proposed region merging strategy, and ② denotes the embedded local search operator.

**Figure 11:**
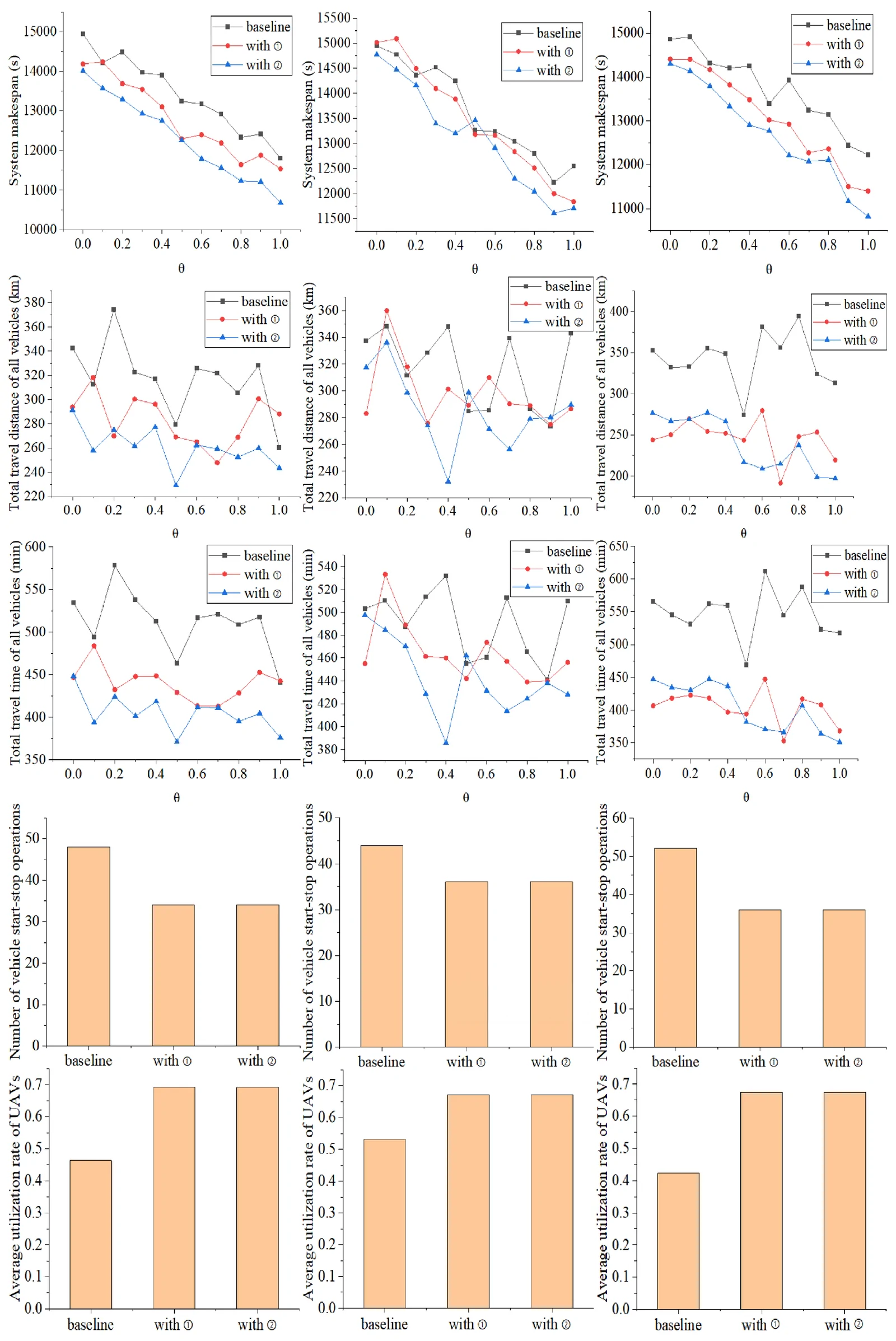
Ablation study results in the Xiangtan study area under the five-vehicle configuration. ① denotes the proposed region merging strategy, and ② denotes the embedded local search operator.

**Figure 12:**
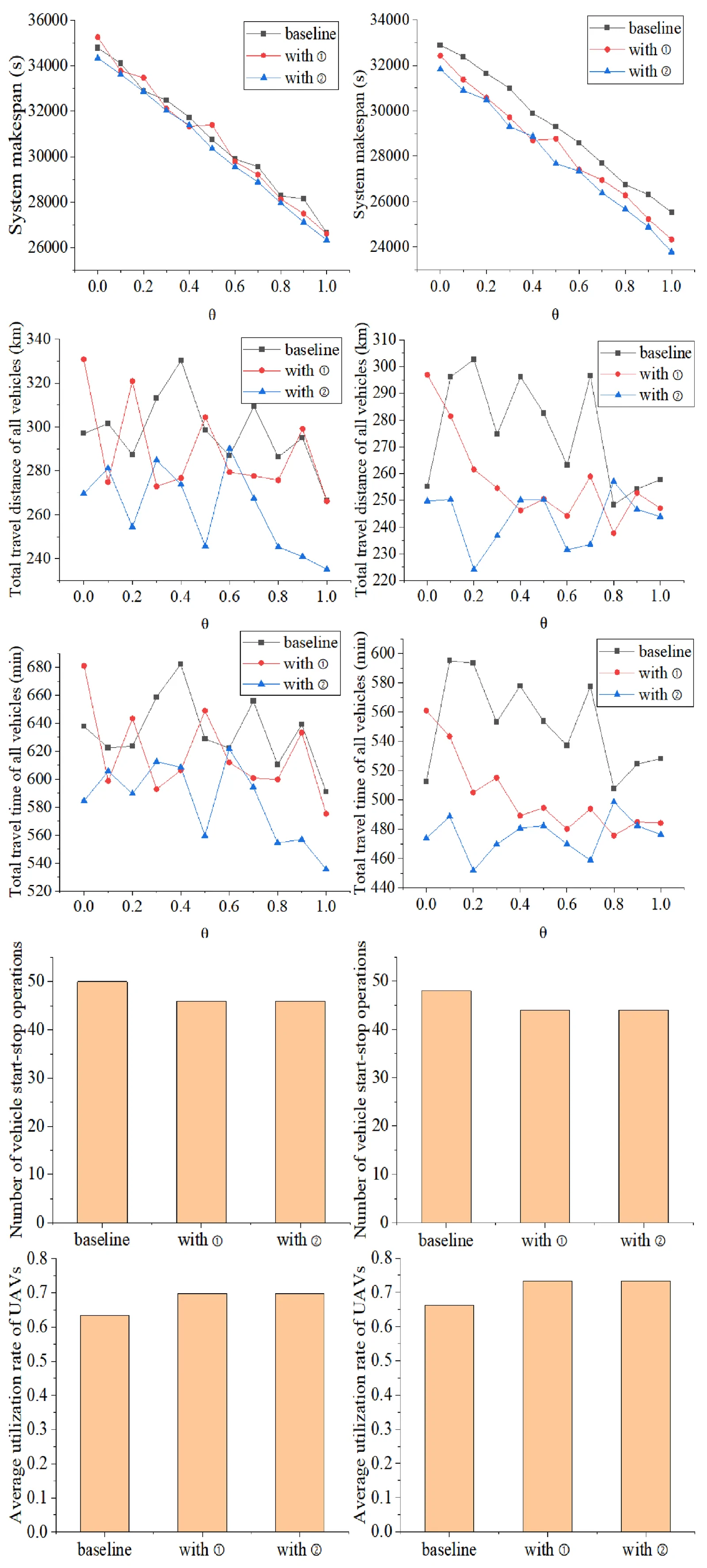
Ablation study results in the Dayao study area under the three-vehicle configuration. ① denotes the proposed region merging strategy, and ② denotes the embedded local search operator.

**Figure 13:**
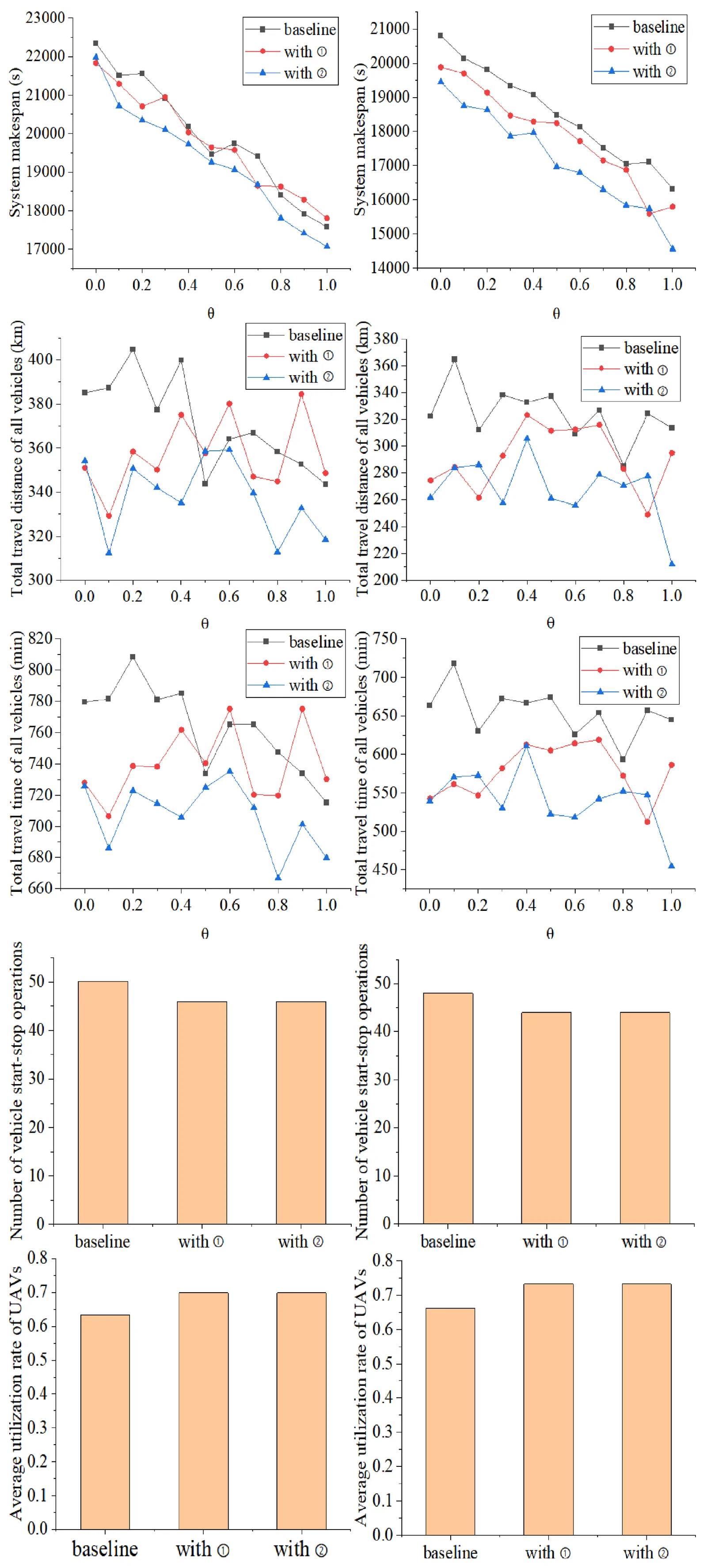
Ablation study results in the Dayao study area under the five-vehicle configuration. ① denotes the proposed region merging strategy, and ② denotes the embedded local search operator.

Figure 10 and Figure 11 present the ablation results for the Xiangtan study area under the three- and five-vehicle configurations, respectively, whereas Figure 12 and Figure 13 present the corresponding results for the Dayao study area. In each figure, the baseline is compared with the result obtained after applying the region merging strategy (①) and that obtained after further introducing the embedded local search operator (②). Overall, region merging mainly reduces vehicle travel distance, travel time, and start-stop operations while increasing average UAV utilization. On this basis, the local search operator further reduces the system makespan by refining the visiting sequence without changing the task-region partition. The consistent trends across the two study areas and vehicle configurations show that the two components improve task organization and scheduling efficiency from complementary perspectives.

Each column in Figure 10, Figure 11, Figure 12 and Figure 13 corresponds to one traffic period, namely one experimental case. Therefore, Figure 10 and Figure 11 contain three columns because three traffic periods were evaluated in the Xiangtan study area, whereas Figure 12 and Figure 13 contain two columns because two traffic periods were evaluated in the Dayao study area. From top to bottom, the five rows present the system makespan, total travel distance of all vehicles, total travel time of all vehicles, number of vehicle start-stop operations, and average UAV utilization rate, respectively. The first three rows plot the corresponding metric against θ, whereas the last two rows use bar charts to directly compare the three method configurations.

As illustrated in Figure 10, Figure 11, Figure 12 and Figure 13 and Table 2, the safety-margin-based region merging strategy substantially reduced vehicle travel costs in the Xiangtan study area. For the three-vehicle configuration, the total travel distance and travel time decreased by an average of 15.59% and 14.85%, respectively. Similar improvements were observed for the five-vehicle configuration, with average reductions of 14.27% and 14.80%. Moreover, the number of vehicle stops was reduced by approximately 12, while the average UAV utilization increased by 20.76%. A similar trend can be observed in the Dayao study area despite its more fragmented parcel distribution. Under the three-vehicle configuration, the proposed merging strategy reduced travel distance and travel time by 4.30% and 5.51%, respectively, while decreasing vehicle stops by four and increasing UAV utilization by 6.76%. For the five-vehicle configuration, the corresponding improvements reached 6.72%, 7.25%, four stops, and 6.76%, respectively. These results demonstrate that the proposed post-processing merging strategy effectively improves the spatial compactness of task regions while reducing unnecessary vehicle transfers. The improvement is particularly pronounced in the Xiangtan dataset, where larger parcels create greater opportunities for merging neighboring regions.

Building upon the optimized task regions, the local search operator further refined the visiting sequence of each vehicle without modifying task assignments. Compared with the original genetic algorithm, the proposed local search reduced the maximum completion time by 1.43% and 3.18% for the three- and five-vehicle configurations in Xiangtan, respectively. Similar improvements of 1.34% and 3.27% were obtained in the Dayao study area.

Overall, the two proposed strategies complement each other from different perspectives. The region merging strategy improves global spatial organization by reducing fragmented task regions and unnecessary vehicle relocations, whereas the local search operator performs fine-grained optimization of visiting sequences under fixed task assignments. Their combination leads to consistently shorter completion times across different study areas and traffic conditions.

**Figure 14:**
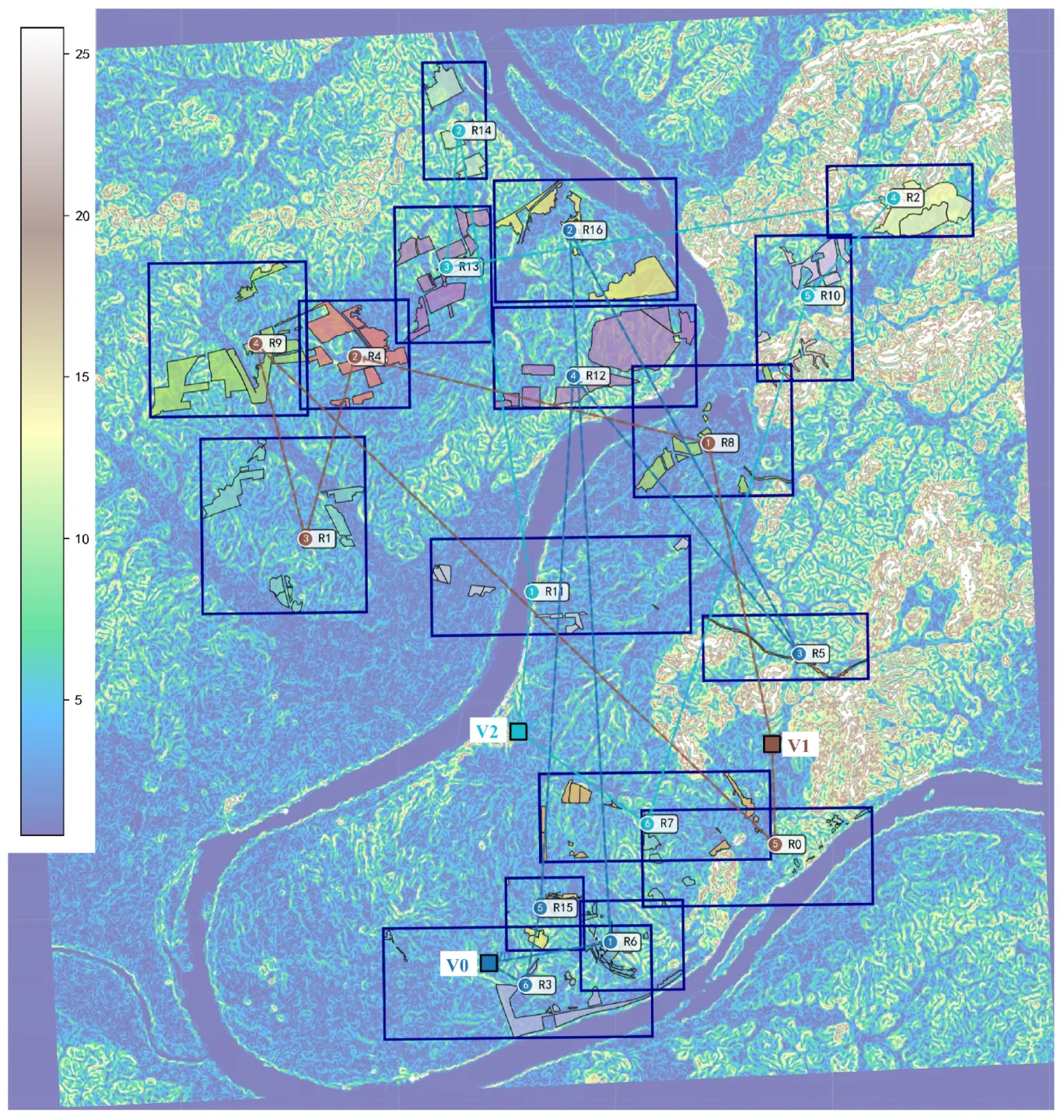
Overall task region partitioning and vehicle–UAV task planning in the Xiangtan study area.

**Figure 15:**
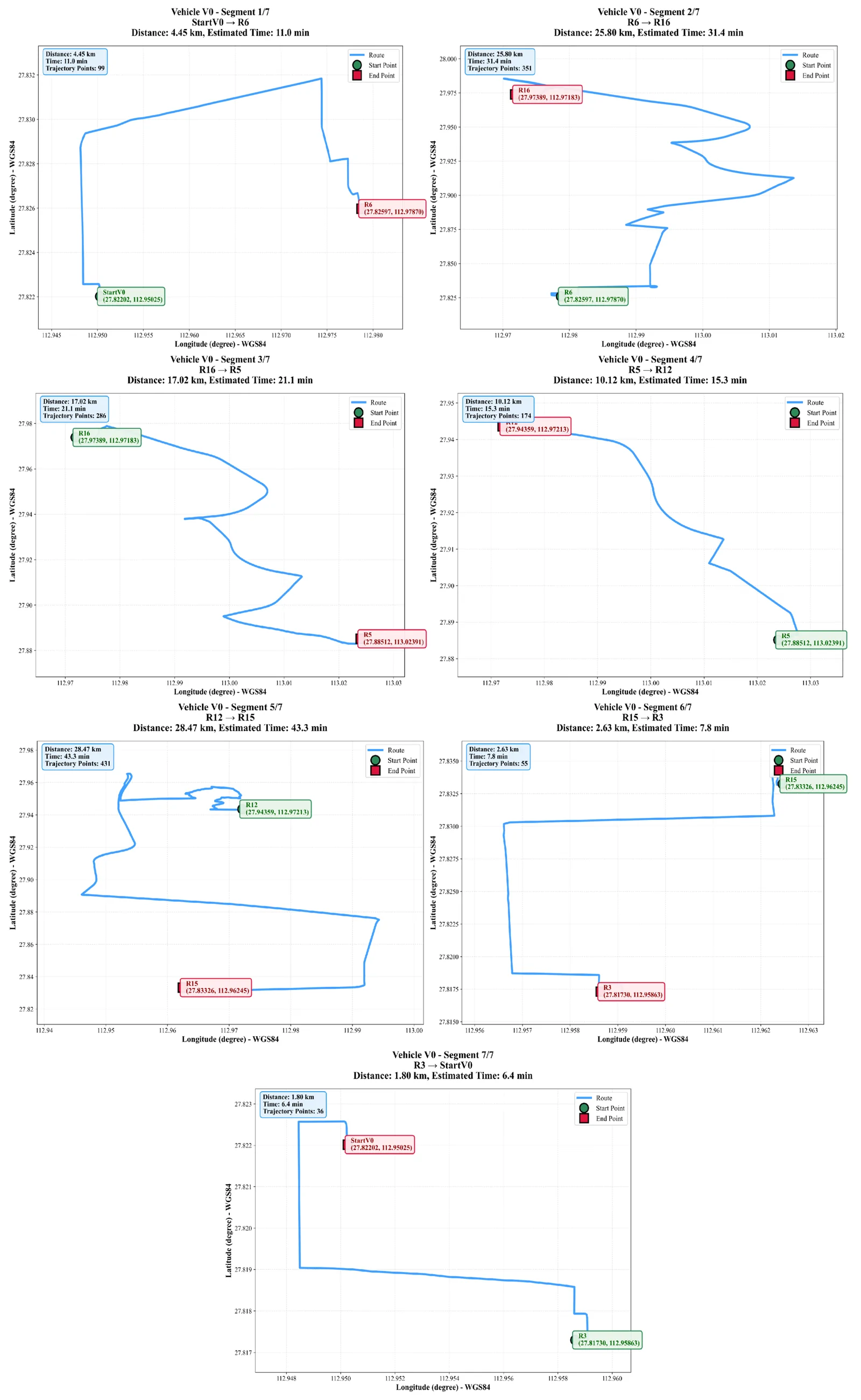
Detailed segmented route of Vehicle $V_{0}$ in the Xiangtan study area.

**Figure 16:**
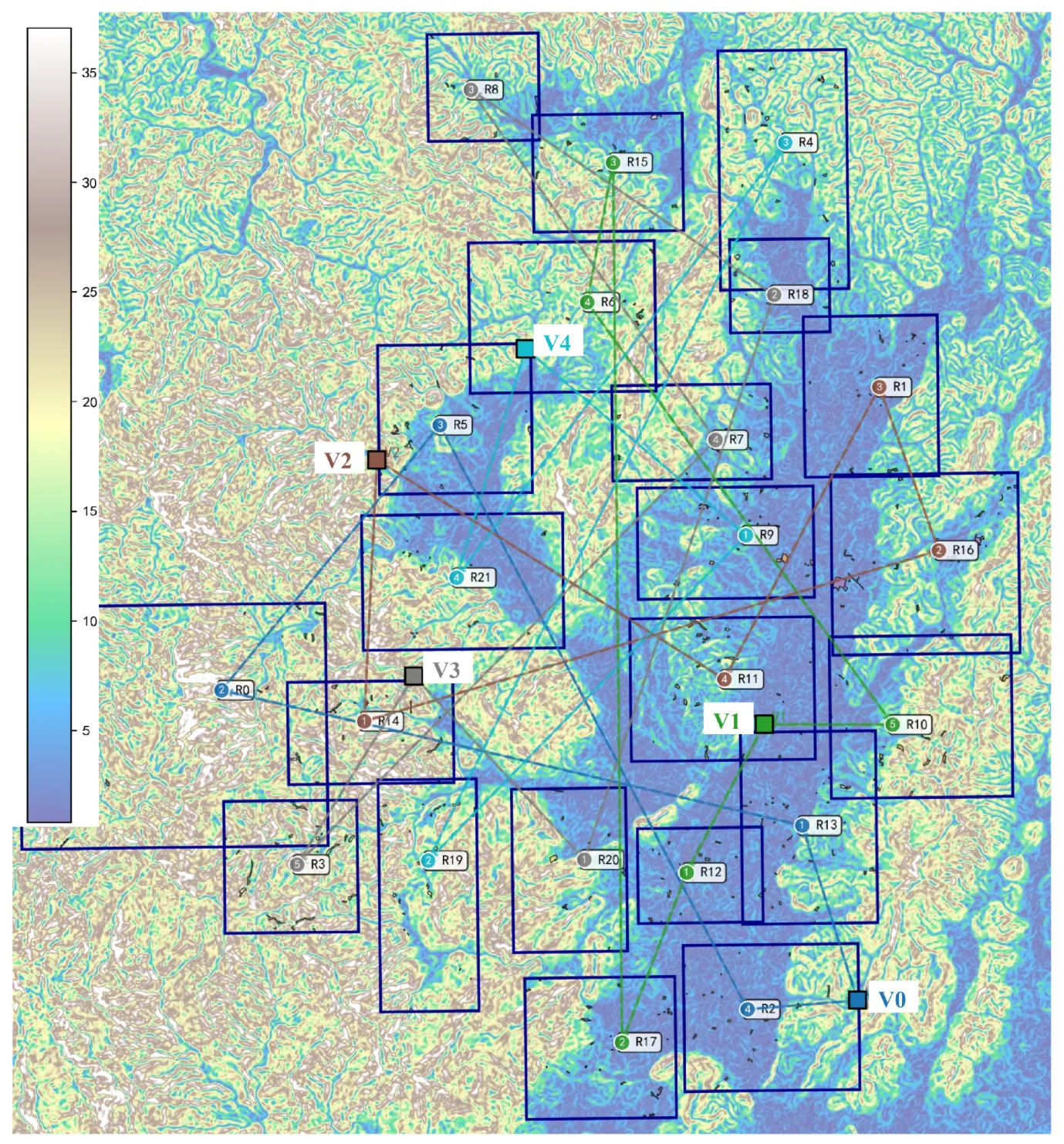
Overall task region partitioning and vehicle–UAV task planning in the Dayao study area.

**Figure 17:**
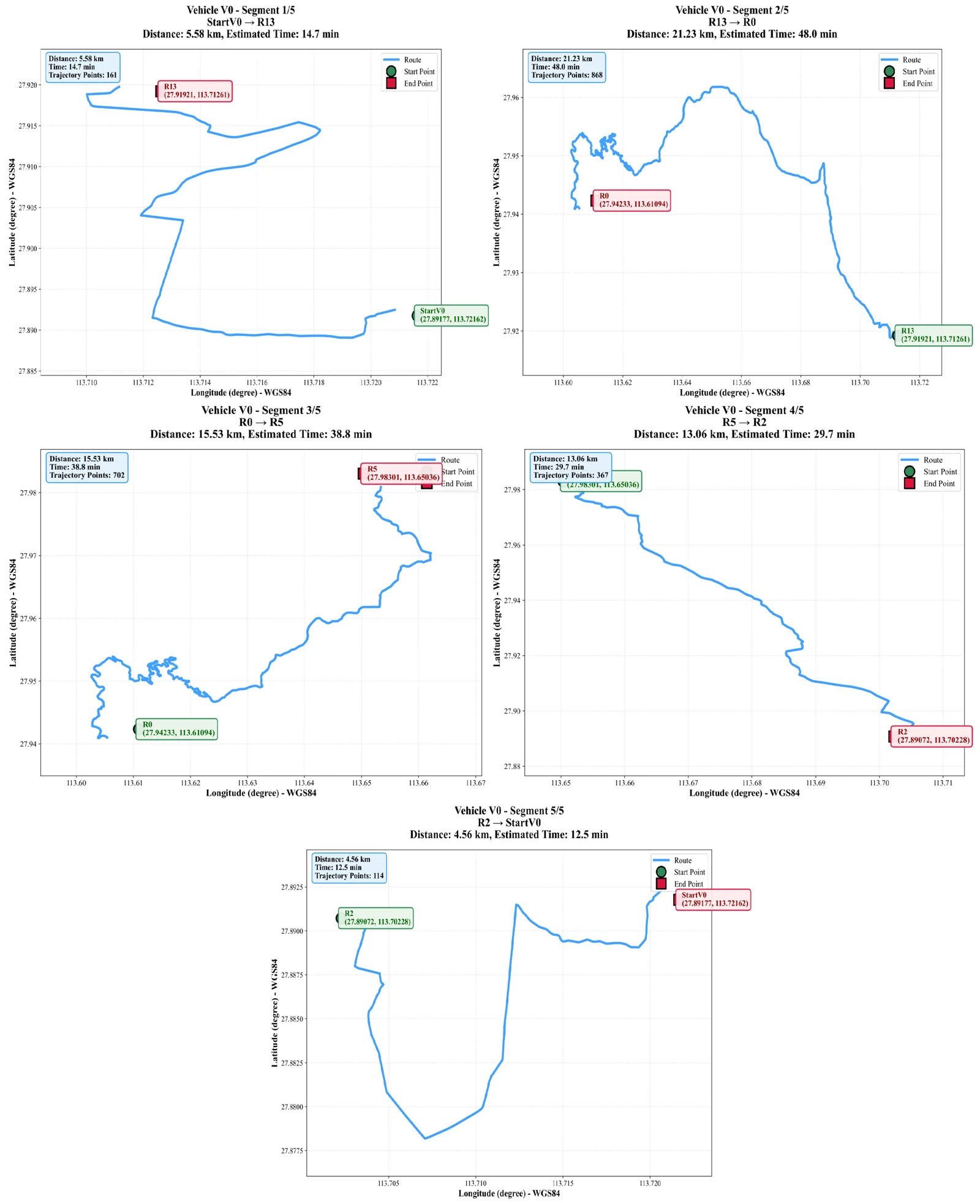
Detailed segmented route of Vehicle $V_{0}$ in the Dayao study area.

Representative scheduling results are visualized in Figure 14, Figure 15, Figure 16 and Figure 17. Figure 14 shows the overall task-region partitioning and the routes assigned to the three vehicle–UAV pairs in the Xiangtan study area, while Figure 16 shows the corresponding results for the five vehicle–UAV pairs in the Dayao study area. In these figures, the labels $V_{i}$ distinguish different vehicle–UAV pairs, the labels $R_{i}$ identify the generated task regions, and the connecting lines indicate the region-visiting sequence assigned to each pair. Figure 15 decomposes the route of Vehicle $V_{0}$ in Xiangtan into seven consecutive segments, whereas Figure 17 presents the five route segments of Vehicle $V_{0}$ in Dayao. For each segment, the travel path, start and end points, travel distance, estimated travel time, and number of trajectory points are provided. Together, these figures show how the generated task regions are assigned and connected into executable vehicle routes.

#### 4.2.2. Workload Comparison of Vehicle–UAV Pairs

Table 3 compares the best, average, and worst pair-level results for all experimental scenarios in terms of parcel count, covered area, vehicle and UAV travel distances, vehicle driving time, UAV operation time, and total completion time. The difference between the shortest and longest pair-level completion times ranged from 0.3 to 11.6 min. Increasing the number of vehicle–UAV pairs from three to five reduced the average covered area per pair from 6.820 to 4.092 km^2^ in Xiangtan and from 0.252 to 0.151 km^2^ in Dayao, while reducing the mean pair-level completion time by 37.5–39.3%. Across all scenarios, the average UAV flight distance (77.2–167.4 km) exceeded the average vehicle driving distance (54.8–91.3 km), and the average UAV operation time was 1.0–48.6% longer than the average vehicle driving time. These results indicate that UAV operation was generally the larger time component, although vehicle driving time remained comparable in some fragmented scenarios.

#### 4.2.3. Comparative Experiments

To further evaluate the optimization capability of DC-GA, four representative metaheuristic algorithms, namely Particle Swarm Optimization (PSO) [30,31], Ant Colony Optimization (ACO), Sparrow Search Algorithm (SSA) [32], and the hybrid SA-SSA algorithm [16], were selected as benchmark methods. For fairness, all algorithms employed the same dual-channel encoding scheme and fitness function.

In addition, the deterministic Nearest Neighbor (NN) method was used as a non-iterative baseline. It assigns the nearest unvisited region to the vehicle with the shortest current completion time. NN provides a simple reference for evaluating the global optimization capability of heuristic methods.

**Figure 18:**
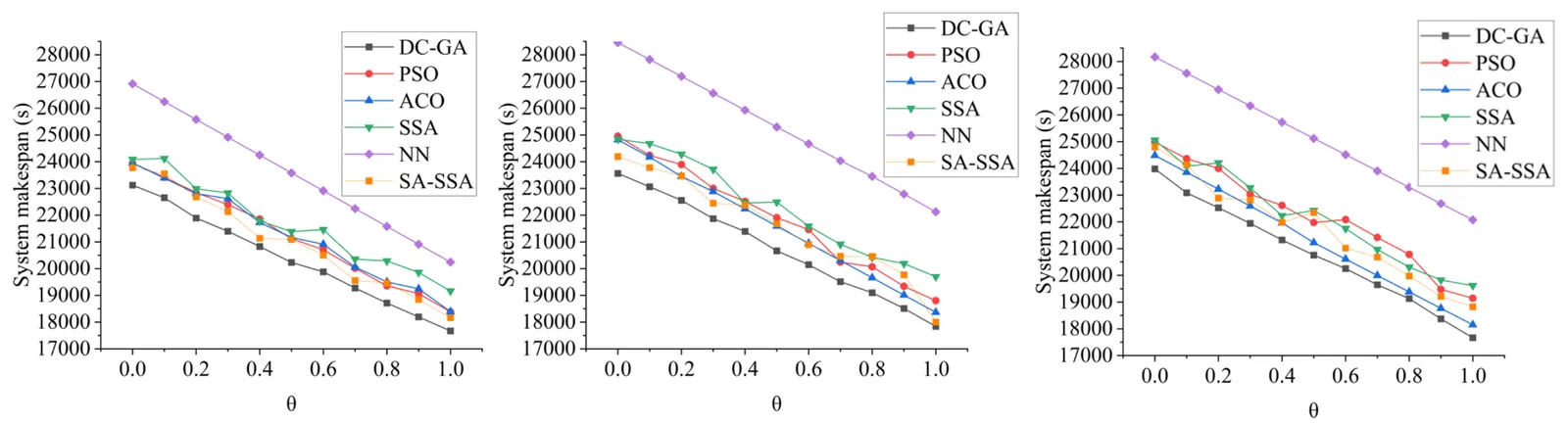
Comparative experiments results in the Xiangtan study area under the three-vehicle configuration.

**Figure 19:**
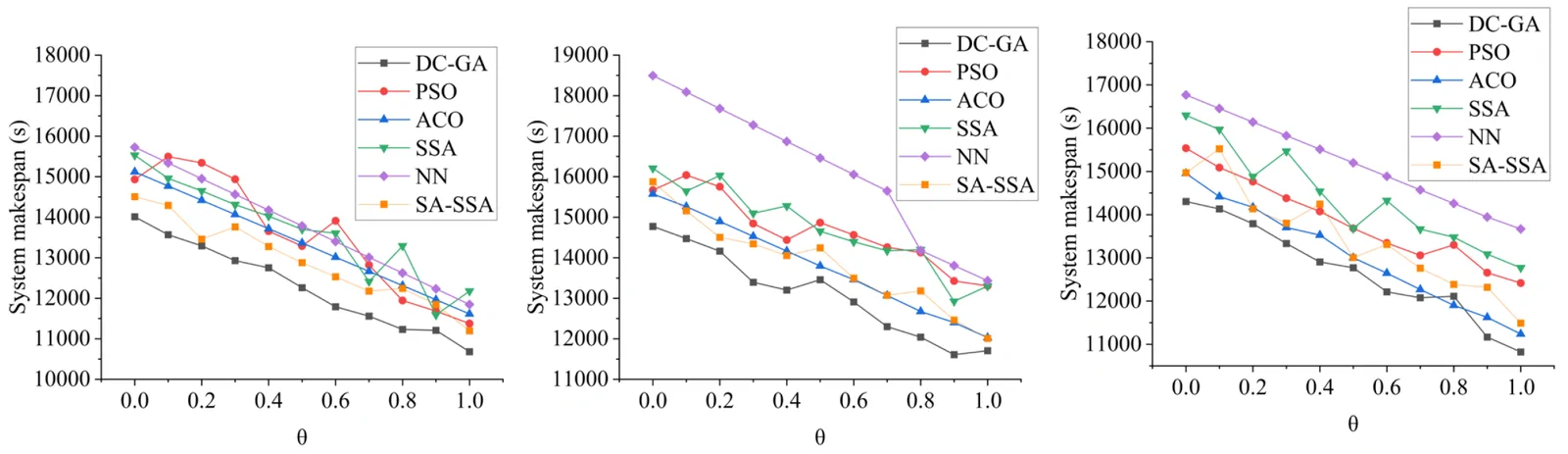
Comparative experiments results in the Xiangtan study area under the five-vehicle configuration.

**Figure 20:**
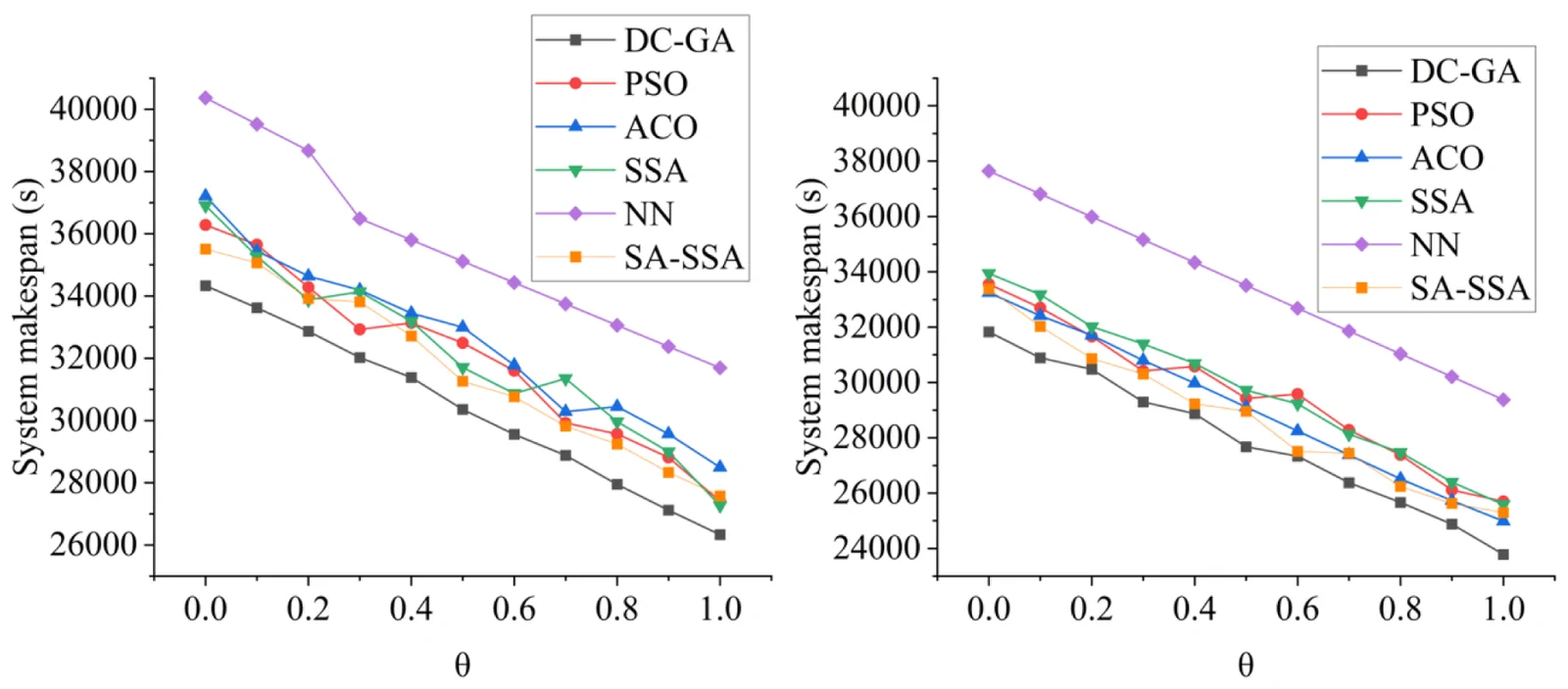
Comparative experiments results in the Dayao study area under the three-vehicle configuration.

**Figure 21:**
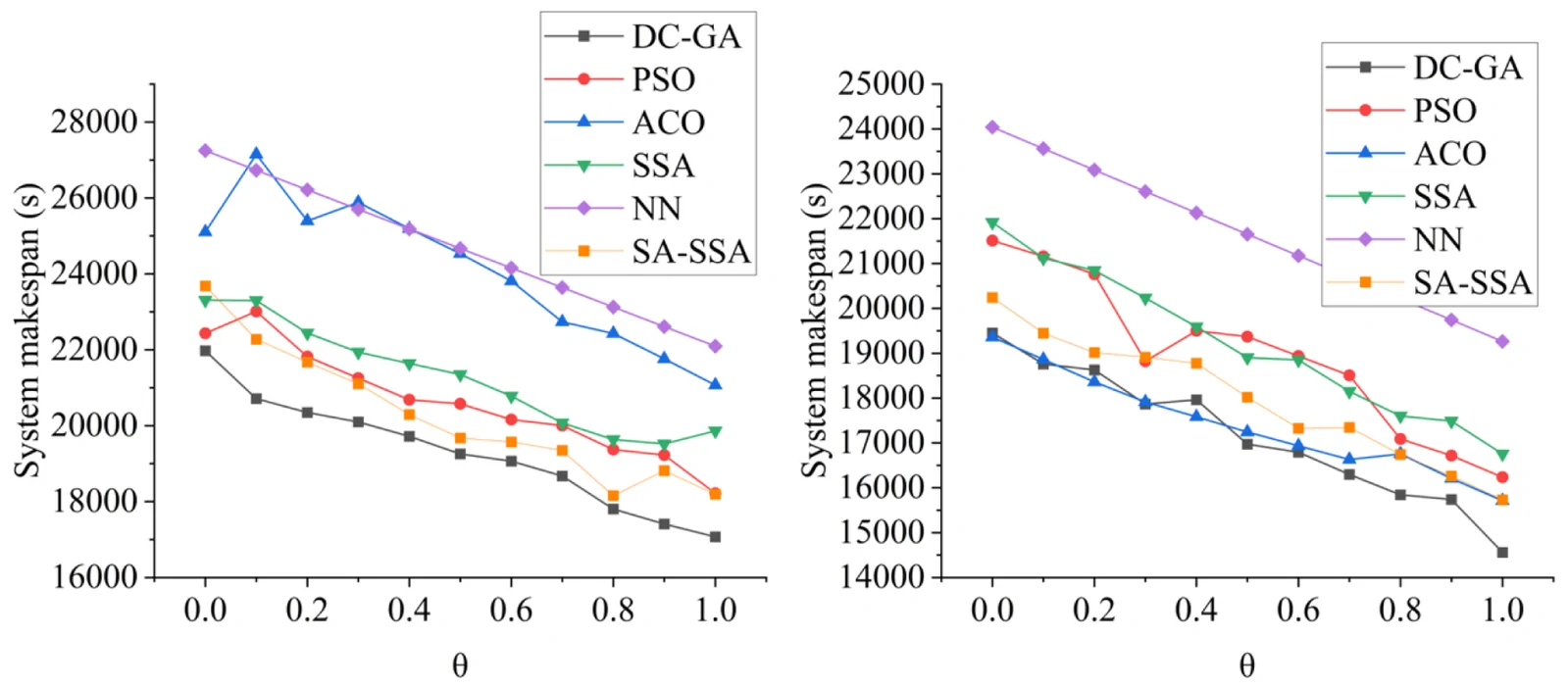
Comparative experiments results in the Dayao study area under the five-vehicle configuration.

The comparison results are presented in Figure 18, Figure 19, Figure 20 and Figure 21 and Table 4. DC-GA consistently achieved the best scheduling performance across all experimental scenarios. For the Xiangtan study area with three vehicles, DC-GA outperformed PSO, ACO, SSA, and SA-SSA by 5.11%, 3.47%, 6.46%, and 3.73%, respectively. Under the five-vehicle configuration, the average improvements further increased to 9.46%, 5.25%, 10.90%, and 5.31%. Similar observations can be made in the Dayao study area. With three vehicles, DC-GA achieved average improvements of 5.34%, 5.39%, 5.85%, and 3.50% over PSO, ACO, SSA, and SA-SSA, respectively. For five vehicles, the corresponding improvements reached 7.92%, 10.72%, 10.00%, and 4.63%.

To evaluate the stability of the stochastic algorithms, DC-GA, PSO, ACO, SSA, and SA-SSA were independently executed 30 times at θ=0.0 for the six Xiangtan configurations. The same seeds were used for all five algorithms in each configuration. NN was excluded from the repeated-run analysis because it is deterministic.

The experimental results are presented in Table 5. The two-sided Wilcoxon signed-rank test was used because the results were paired by random seed and no normality assumption was required. DC-GA was separately compared with PSO, ACO, SSA, and SA-SSA in each configuration. The Holm method was applied to the four pairwise *p*-values to control the effect of multiple comparisons. All adjusted *p*-values remained below 0.05. The largest adjusted value was $8.33\times 10^{-7}$. DC-GA also achieved the lowest mean makespan in all six configurations. These results confirm that the performance advantage of DC-GA is statistically significant and is not caused by random initialization.

For each of the six configurations, the rankings of the five stochastic algorithms based on the best- and worst-run makespans were consistent with the ranking based on the mean values.

For a representative comparison at θ=0.0, NN produced a makespan of 28,453.8 s in Case 2 of the Xiangtan study area under the three-vehicle configuration. In the same case, the mean makespans of DC-GA, PSO, ACO, SSA, and SA-SSA over 30 independent runs were 23,658.9, 25,092.3, 24,772.2, 25,213.6, and 24,413.6 s, respectively. Thus, the metaheuristic algorithms reduced the makespan by 11.39–16.85% compared with NN, with DC-GA achieving the largest reduction.

The computational cost of the deterministic scheduling stage was also evaluated under the same computing environment. Across the six Xiangtan configurations, the overall average execution times of DC-GA, PSO, ACO, SSA, and SA-SSA were 59.4, 18.7, 34.3, 22.4, and 22.6 s, respectively. NN required only 0.0034 s on average because it did not involve iterative optimization. DC-GA had the highest computational cost. This additional cost mainly resulted from the local search performed after population initialization and genetic operations. Nevertheless, the optimization is conducted offline before mission execution. The runtime is, therefore, acceptable for the pre-mission scheduling considered in this study.

**Figure 22:**
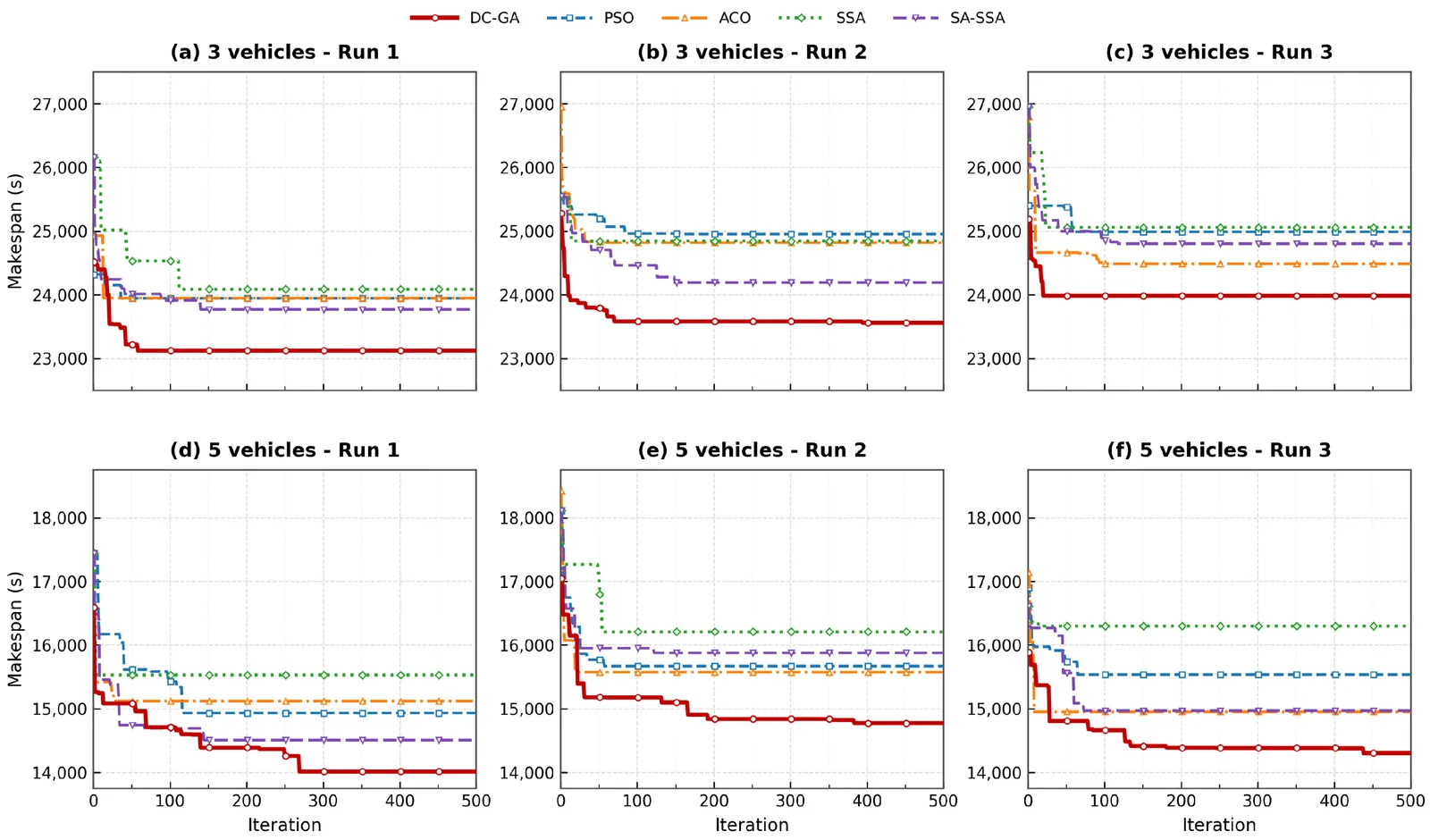
Convergence curves of different methods in the Xiangtan study area under the three- and five-vehicle configurations.

**Figure 23:**
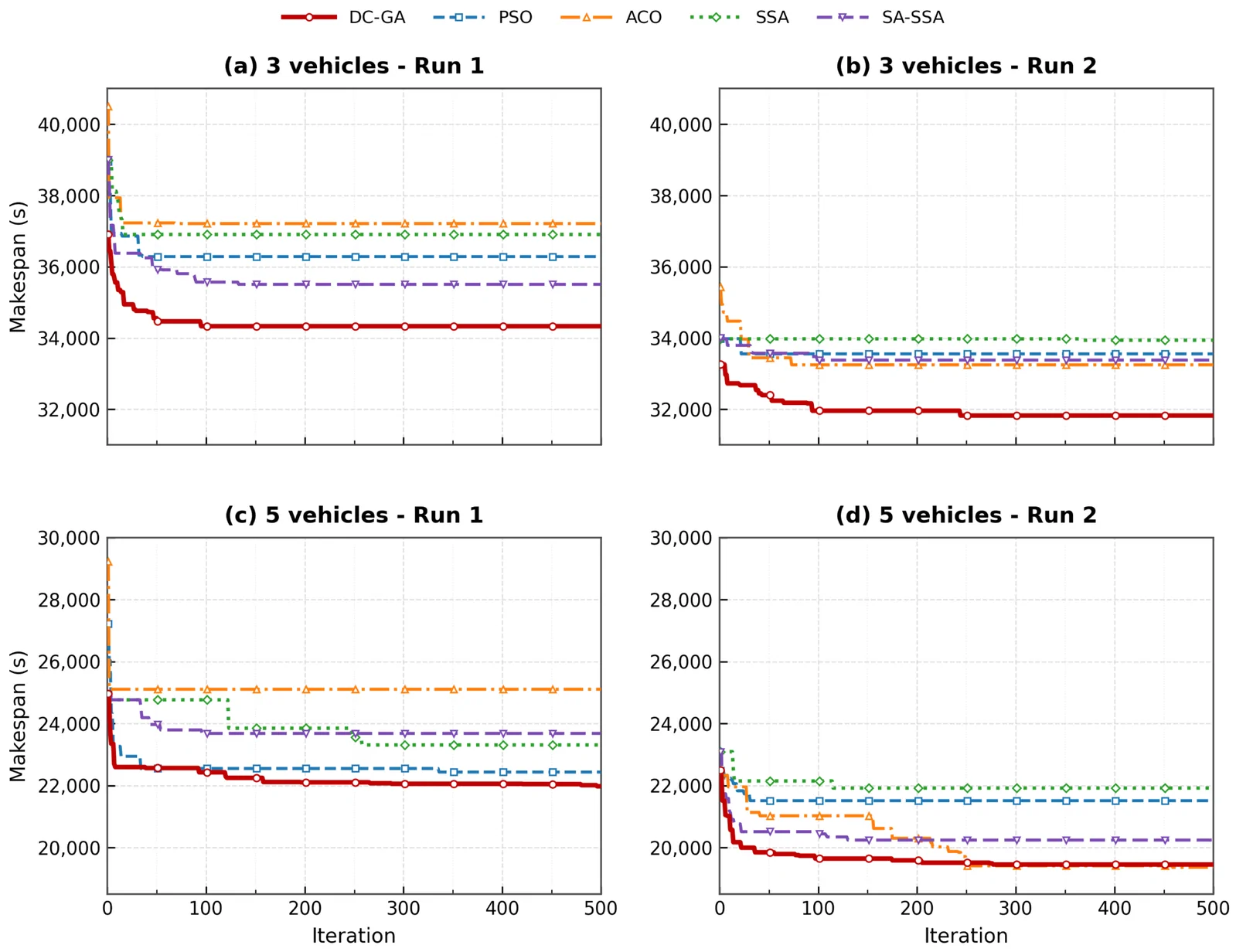
Convergence curves of different methods in the Dayao study area under the three- and five-vehicle configurations.

To further examine the search dynamics, Figure 22 and Figure 23 present the best-so-far makespan over 500 iterations at θ=0.0. DC-GA rapidly reduced the makespan during the early iterations in both study areas. It also continued to improve after most benchmark algorithms had stabilized. Across the tested cases, DC-GA converged to the lowest or a comparable final makespan. These results demonstrate that DC-GA provides both effective early-stage search and stable subsequent optimization.

#### 4.2.4. Performance of the Proposed UATSF

To validate the effectiveness of the proposed UATSF under stochastic operating conditions, three scheduling strategies were compared: (1) Worst-case scheduling, which optimizes using the upper-bound operation times; (2) Best-case scheduling, which optimizes using the lower-bound operation times; (3) The proposed UATSF, which automatically determines the optimal uncertainty parameter θ through parameter scanning and evaluates solution robustness via Monte Carlo simulation.

The experimental results are summarized in Table 6 and Table 7. The best-case strategy achieved the smallest planned completion time because it assumes the minimum operation time for every task region. However, none of the generated schedules satisfied the 95% reliability requirement under stochastic simulations, resulting in a 0% success rate across all test scenarios. This indicates that optimizing solely under best-case assumptions is insufficient for practical UAV inspection missions in complex terrain, where operation times are inherently uncertain. Conversely, the worst-case strategy guaranteed 100% reliability in every scenario by assuming the maximum operation time for all task regions. Although this conservative strategy ensured robust execution, it significantly increased the planned completion time and reduced overall resource utilization.

Compared with these two baselines, the proposed UATSF achieved a substantially better balance between scheduling efficiency and robustness. Across all experimental scenarios, the reliability of UATSF ranged from 86.20% to 98.40%, with most cases remaining close to the target reliability level of 95%. Meanwhile, its planned completion time was consistently shorter than that of the worst-case strategy. In the Xiangtan study area, UATSF reduced the planned completion time by approximately 6.80–11.09% on average while maintaining comparable reliability. Similar improvements were observed in the Dayao study area.

These results indicate that the automatically identified uncertainty parameter approximates the modeled uncertainty level under the assumed simulation settings. Within these settings, the deterministic operation times generated by the corresponding θ provide a surrogate for the simulated robust completion time, thereby avoiding the excessive conservatism of worst-case optimization while achieving high reliability under the modeled uncertainty.

The scanning step size was evaluated using τ=0.1 and τ=0.05 in the six Xiangtan cases. All other experimental settings were unchanged. Table 8 summarizes the results. Reducing τ increased the number of candidate θ values from 11 to 21. The average completion rate increased from 93.47% to 97.17%. The average maximum system completion time increased from 17,170.6 s to 17,440.7 s, corresponding to an increase of 1.59%. Thus, a finer step provides a more conservative schedule and a higher empirical completion rate. It also requires nearly twice as many parameter evaluations. The default value τ=0.1 was retained as a practical balance between scanning resolution and computational cost.

## 5. Discussion

The experimental results show that the three components address different operational bottlenecks. CTRG reduces unnecessary vehicle transfers by aggregating spatially adjacent parcels under UAV capability constraints, while DC-GA jointly improves task allocation and region visiting sequences. UATSF further avoids the excessive conservatism of worst-case scheduling while achieving a simulated reliability of 86.20–98.40% under the modeled uncertainty. In practice, the framework can support pre-mission planning for vehicle–UAV field verification in natural resource surveys: parcel locations, road travel times, terrain data, and UAV parameters are used to generate executable task regions and coordinated vehicle routes.

The present framework focuses on pre-mission scheduling and uses terrain information to distinguish the relative level of operation-time uncertainty within each mission. Within this scope, the data-adaptive risk categories and consistently defined uncertainty parameters provide a common basis for comparing alternative scheduling solutions. These parameters are mission-oriented engineering modeling settings rather than universal physical constants and should be recalibrated when the method is transferred to other UAV platforms or operating environments. The reported results were obtained using the specific settings adopted in this study, including η=0.8, ρ=0.95, the spatial proximity threshold $d_{ij}\leq 2D_{\max}$, and the scanning step τ=0.1. The numerical results may vary under substantially different configurations. Future work will combine flight logs with meteorological and positioning data to establish more transferable risk thresholds and uncertainty distributions. The framework will also be extended toward online replanning, fully stochastic optimization, and parcel-specific image-acquisition requirements to support more dynamic and diverse field operations.

The current framework adopts several modeling abstractions to maintain the tractability of pre-mission scheduling. The linear charging model is used to estimate whether vehicle travel can cover the charging demand between consecutive tasks. Its purpose is to support scheduling decisions rather than to reproduce detailed battery electrochemical processes. The stochastic operation time partly reflects the additional flight duration caused by wind disturbances and attitude adjustments. Future work will combine flight logs with battery state-of-charge and temperature data. This will allow nonlinear charging, battery degradation, and thermal effects to be modeled more accurately.

The geometric center provides a consistent spatial reference for task organization and route estimation. Our field investigations indicate that parcels requiring verification are commonly associated with human activities. Their surrounding road accessibility is, therefore, generally favorable. In addition, vehicle travel time is calculated from actual road-network routes. Nevertheless, some geometric centers may not be suitable for vehicle stopping or UAV takeoff. Future work will include the selection of nearby road-accessible stopping points. The UAV route, endurance, and communication constraints will then be updated according to the actual takeoff location.

The current implementation uses the Amap API to obtain road travel times. However, the optimization model only requires a road-network travel-time matrix and is not tied to a specific map provider. Future work will develop a replaceable road-data interface. This will allow Google Maps, OpenStreetMap-based routing, or local road-network data to be used in different regions. The terrain-risk model currently uses DEM-derived slope to characterize the persistent influence of terrain morphology. This provides stable risk information for pre-mission planning. Future work will further combine meteorological information with onboard sensing data. Dynamic conditions such as fog, precipitation, and sudden wind shifts can then be incorporated into risk updating and online replanning.

## 6. Conclusions

This study focuses on large-scale remote sensing monitoring and field verification of natural resources in complex terrain. It addresses the fragmented distribution of target parcels, the coupled allocation and routing of multiple vehicle–UAV pairs, and the uncertainties in vehicle travel and UAV operation times. To address these problems, a spatiotemporal uncertainty-aware cooperative task planning framework integrating CTRG, DC-GA, and UATSF is proposed. Experiments conducted in two real-world study areas under three- and five-vehicle configurations demonstrate the effectiveness of the framework. The CTRG region-merging strategy reduced total vehicle travel distance and travel time by 4.30–15.59% and 5.51–14.85%, respectively, while improving UAV utilization. Compared with representative heuristic algorithms, DC-GA reduced the system makespan by 3.47–10.90%, and its local search mechanism further reduced the mission completion time by 1.34–3.27%. UATSF shortened the planned completion time by 6.80–11.09% compared with worst-case scheduling while achieving a simulated reliability of 86.20–98.40% under the modeled uncertainty. These results show that CTRG improves the spatial organization of fragmented parcels, DC-GA enhances workload coordination and scheduling efficiency, and UATSF provides an effective balance between planning efficiency and completion reliability under spatiotemporal uncertainty.

More broadly, these findings indicate that effective vehicle–UAV cooperation requires spatial task organization, platform capability constraints, and temporal uncertainty to be considered within a unified planning process. They also suggest that combining deterministic optimization with stochastic reliability evaluation provides a useful methodological principle for balancing efficiency and robustness in uncertain field operations.

By reducing unnecessary vehicle relocation and improving the reliability of mission completion, the proposed framework has the potential to increase the number of parcels verified within a limited fieldwork period and to support more timely evidence collection for natural resource surveys and cultivated land protection.

## Figures and Tables

**Table 1:** Comparison of representative studies.

Study	Application	Main Objective	Vehicle–UAV Cooperation	Uncertainty Considered
Ref. [16]	Urban logistics	Vehicle routing performance	No	None
Ref. [17]	Agricultural coverage	Coverage efficiency and path quality	No	None
Ref. [18]	Urban coverage	Cooperative coverage efficiency	UGV-UAV	None
Ref. [19]	3D reconstruction	Acquisition and processing time	vehicle-UAV	None
Ref. [20]	Humanitarian logistics	Cost and worst tardiness penalty	Truck-UAV pairs	Demand and truck travel time
Ref. [21]	Last-mile delivery	Cost and time-window penalties	Truck-UAV pairs	Truck travel time
Ref. [22]	Delivery and pickup	Transportation time and UAV energy	One truck and multiple UAVs	UAV flight speed
Ref. [12]	Natural resource surveys	Mobile sensing and local coverage	Vehicle-UAV	None
This study	Natural resource verification	Makespan and schedule reliability	Multiple vehicle-UAV pairs	Vehicle and UAV operation times

Note: “None” indicates that the corresponding study assumes deterministic operating times.

**Table 2:** Performance improvement under different numbers of vehicle-UAVs at two test sites.

Site	Vehicle-UAVs	Case	Distance	Time	Stops	Utilization
			**Reduction (%)**	**Reduction (%)**	**Reduced**	**Improvement (%)**
Site 1 (Xiangtan)	3	Case 1	16.99	19.84	14	22.98
		Case 2	7.04	6.26	8	14.06
		Case 3	22.73	18.46	16	25.23
		Avg.	15.59	14.85	12.67	20.76
	5	Case 1	9.85	13.51	14	22.95
		Case 2	5.41	5.05	8	14.06
		Case 3	27.54	25.83	16	25.23
		Avg.	14.27	14.80	12.67	20.76
Site 2 (Dayao)	3	Case 1	2.54	2.46	4	6.52
		Case 2	6.05	8.55	4	7.00
		Avg.	4.30	5.51	4.00	6.76
	5	Case 1	3.52	2.98	4	6.52
		Case 2	9.92	11.52	4	7.00
		Avg.	6.72	7.25	4.00	6.76

**Table 3:** Best, average, and worst workload measurements of individual vehicle–UAV pairs under all experimental scenarios.

(a) Task-Scale and Distance Measurements						
Site	Vehicle–UAVs	Case	Parcels	Covered Area	Vehicle Distance	UAV Distance
				(km^2^)	(km)	(km)
Site 1 (Xiangtan)	3	Case 1	49/48.3/50	7.231/6.820/6.554	90.3/80.9/85.1	129.1/128.6/128.2
		Case 2	55/48.3/47	7.681/6.820/7.832	102.4/87.1/80.5	131.9/132.1/129.5
		Case 3	40/48.3/52	6.078/6.820/8.372	71.2/70.8/75.5	134.0/132.9/131.3
	5	Case 1	26/29/43	5.617/4.092/3.002	42.7/55.4/54.1	81.2/77.2/78.5
		Case 2	17/29/41	4.241/4.092/3.825	57.4/54.8/55.1	77.0/79.3/82.2
		Case 3	24/29/34	4.590/4.092/3.964	46.4/55.4/52.9	81.9/79.8/83.6
Site 2 (Dayao)	3	Case 1	187/159.3/120	0.307/0.252/0.213	86.0/91.3/107.7	182.7/166.7/135.1
		Case 2	180/159.3/135	0.232/0.252/0.198	69.3/83.4/103.2	171.6/167.4/166.2
	5	Case 1	86/95.6/79	0.103/0.151/0.137	86.9/67.0/79.8	90.0/100.0/94.7
		Case 2	85/95.6/90	0.158/0.151/0.120	84.6/61.1/60.0	85.0/100.5/105.2
**(b) Time Measurements**						
**Site**	**Vehicle–UAVs**	**Case**	**Vehicle Time**	**UAV Operation Time**	**Completion Time**	
			**(min)**	**(min)**	**(min)**	
Site 1 (Xiangtan)	3	Case 1	136.3/125.6/115.8	175.3/174.2/173.9	346.8/346.9/347.1	
		Case 2	131.6/130.8/137.9	180.0/178.9/175.3	353.3/355.1/356.5	
		Case 3	105.5/123.6/139.5	180.0/179.9/178.9	353.4/354.3/355.4	
	5	Case 1	78.7/83.7/70.1	108.4/104.5/108.3	208.6/210.5/212.6	
		Case 2	94.6/85.7/67.5	106.2/110.7/116.4	219.1/221.0/223.2	
		Case 3	74.8/89.5/78.2	113.8/111.4/117.0	220.7/221.6/222.2	
Site 2 (Dayao)	3	Case 1	150.2/202.9/288.6	261.8/237.7/192.2	518.0/521.3/523.1	
		Case 2	132.6/160.3/189.1	246.0/238.2/233.8	479.8/480.7/481.2	
	5	Case 1	148.2/141.1/173.5	128.2/142.6/134.0	321.0/325.4/328.7	
		Case 2	151.7/122.2/143.7	123.0/142.9/147.5	287.8/295.8/299.4	

Note: Numerical entries are ordered as best/average/worst (B/A/W). Total completion time also includes preparation and charging-wait time.

**Table 4:** Comparison results.

Site	Vehicle-UAVs	Case	vs. PSO (%)	vs. ACO (%)	vs. SSA (%)	vs. SA-SSA (%)
Site 1 (Xiangtan)	3	Case 1	3.97	4.25	6.13	3.03
		Case 2	5.05	3.82	7.00	3.93
		Case 3	6.30	2.34	6.25	4.23
		Avg.	5.11	3.47	6.46	3.73
	5	Case 1	9.17	7.99	9.89	4.88
		Case 2	10.79	5.12	11.08	5.47
		Case 3	8.42	2.63	11.74	5.58
		Avg.	9.46	5.25	10.90	5.31
Site 2 (Dayao)	3	Case 1	5.00	6.74	5.39	3.91
		Case 2	5.67	4.04	6.31	3.09
		Avg.	5.34	5.39	5.85	3.50
	5	Case 1	6.47	19.91	9.33	4.70
		Case 2	9.37	1.52	10.67	4.56
		Avg.	7.92	10.72	10.00	4.63

**Table 5:** Statistical results of 30 independent runs at θ=0.0 in the Xiangtan study area.

Vehicle–UAVs	Case	DC-GA	PSO	ACO	SSA	SA-SSA	Max. Adjusted $p_{\mathrm{adj}}^{\max}$
3	Case 1	23,094.8 ± 102.2	24,284.2 ± 343.0	23,827.2 ± 305.1	24,500.1 ± 351.6	23,824.3 ± 251.6	$7.45\times 10^{-9}$
	Case 2	23,658.9 ± 90.4	25,092.3 ± 336.6	24,772.2 ± 100.8	25,213.6 ± 381.0	24,413.6 ± 331.5	$7.45\times 10^{-9}$
	Case 3	23,741.6 ± 115.3	24,986.7 ± 314.5	24,484.9 ± 43.7	25,288.0 ± 331.5	24,567.2 ± 318.6	$7.45\times 10^{-9}$
5	Case 1	13,945.2 ± 175.1	15,249.3 ± 448.3	15,084.7 ± 131.4	15,415.1 ± 318.7	14,485.2 ± 246.2	$8.33\times 10^{-7}$
	Case 2	14,658.8 ± 250.9	16,144.1 ± 430.9	15,553.2 ± 221.6	16,278.8 ± 356.3	15,327.6 ± 267.0	$7.45\times 10^{-9}$
	Case 3	14,362.8 ± 160.2	15,674.5 ± 482.3	14,841.1 ± 83.2	16,007.6 ± 321.8	15,055.7 ± 375.6	$3.54\times 10^{-8}$

Note: Makespan values are reported as mean ± standard deviation in seconds. $p_{\mathrm{adj}}^{\max}$ denotes the largest Holm-adjusted *p*-value among the four pairwise Wilcoxon signed-rank tests comparing DC-GA with PSO, ACO, SSA, and SA-SSA.

**Table 6:** Comparison of completion rate and maximum system completion time under different methods.

Site	Vehicle-UAVs	Case	Method (1)		Method (2)		Method (3)	
			Completion Rate (%)	Max. System Completion Time (s)	Completion Rate (%)	Max. System Completion Time (s)	Completion Rate (%)	Max. System Completion Time (s)
Site 1 (Xiangtan)	3	Case 1	100	23,120.6	0	17,671.0	96.30	20,828.3
		Case 2	100	23,560.9	0	17,839.1	94.90	21,392.5
		Case 3	100	23,982.6	0	17,667.8	96.80	21,323.3
	5	Case 1	100	14,011.9	0	10,680.8	86.20	12,753.2
		Case 2	100	14,774.1	0	11,705.5	93.40	13,394.9
		Case 3	100	14,303.7	0	10,822.3	93.20	13,331.6
Site 2 (Dayao)	3	Case 1	100	34,332.3	0	26,331.2	98.40	31,383.5
		Case 2	100	31,825.3	0	23,784.3	95.60	28,872.4
	5	Case 1	100	21,975.0	0	17,069.0	87.20	19,720.0
		Case 2	100	19,452.3	0	14,560.2	91.80	17,963.8

**Table 7:** Comparison of completion time variation under Method (3).

Site	Vehicle-UAVs	Case	Completion Time Reduction (%)	Completion Time Increase (%)
Site 1 (Xiangtan)	3	Case 1	9.91	15.16
		Case 2	9.20	16.61
		Case 3	11.09	17.14
	5	Case 1	8.98	16.25
		Case 2	9.34	12.61
		Case 3	6.80	18.82
Site 2 (Dayao)	3	Case 1	8.59	16.10
		Case 2	9.28	17.62
	5	Case 1	10.26	13.44
		Case 2	7.65	18.95

**Table 8:** Sensitivity analysis of the scanning step size in the Xiangtan study area.

Vehicle–UAVs	Case	τ=0.1		τ=0.05	
		Completion Rate (%)	Max. System Completion Time (s)	Completion Rate (%)	Max. System Completion Time (s)
3	Case 1	96.30	20,828.3	98.90	21,105.8
	Case 2	94.90	21,392.5	96.10	21,647.3
	Case 3	96.80	21,323.3	98.70	21,740.4
	Avg.	96.00	21,181.4	97.90	21,497.8
5	Case 1	86.20	12,753.2	90.30	12,875.3
	Case 2	93.40	13,394.9	99.20	13,711.8
	Case 3	93.20	13,331.6	99.80	13,563.7
	Avg.	90.93	13,159.9	96.43	13,383.6

## Data Availability

The raw data supporting the conclusions of this article will be made available by the authors on request. The code for data analysis is available upon request from the authors.
